# Porous Microspheres Comprising CoSe_2_ Nanorods Coated with N-Doped Graphitic C and Polydopamine-Derived C as Anodes for Long-Lived Na-Ion Batteries

**DOI:** 10.1007/s40820-022-00855-z

**Published:** 2022-04-28

**Authors:** Jae Seob Lee, Rakesh Saroha, Jung Sang Cho

**Affiliations:** Department of Engineering Chemistry, Chungbuk National University, Cheongju, Chungbuk 361-763 Republic of Korea

**Keywords:** Spray pyrolysis, Sodium-ion batteries, Cobalt selenide nanorods, Porous microspheres, Dual carbon coating

## Abstract

Metal–organic framework-templated nitrogen-doped graphitic carbon (NGC) and polydopamine-derived carbon (PDA-derived C)-double coated one-dimensional CoSe_2_ nanorods supported highly porous three-dimensional microspheres are introduced as anodes for excellent Na-ion batteries, particularly with long-lived cycle under carbonate-based electrolyte system. The microspheres uniformly composed of ZIF-67 polyhedrons and polystyrene nanobeads (*ϕ* = 40 nm) are synthesized using the facile spray pyrolysis technique, followed by the selenization process (P-CoSe_2_@NGC NR). Further, the PDA-derived C-coated microspheres are obtained using a solution-based coating approach and the subsequent carbonization process (P-CoSe_2_@PDA-C NR). The rational synthesis approach benefited from the synergistic effects of dual carbon coating, resulting in a highly conductive and porous nanostructure that could facilitate rapid diffusion of charge species along with efficient electrolyte infiltration and effectively channelize the volume stress. Consequently, the prepared nanostructure exhibits extraordinary electrochemical performance, particularly the ultra-long cycle life stability. For instance, the advanced anode has a discharge capacity of 291 (1000th cycle, average capacity decay of 0.017%) and 142 mAh g^−1^ (5000th cycle, average capacity decay of 0.011%) at a current density of 0.5 and 2.0 A g^−1^, respectively.

**Supplementary Information:**

The online version contains supplementary material available at 10.1007/s40820-022-00855-z.

## Highlights

- One-dimensional CoSe_2_ nanorods supported on three-dimensional microspheres were prepared via spray pyrolysis.
- Nanorods were coated by N-doped graphitic C and polydopamine-derived C.
- The unique nanostructure exhibits exceptional cycling stability (5000 cycles at 2.0 A g^−1^).

**Supplementary Information:**

The online version contains supplementary material available at 10.1007/s40820-022-00855-z.

## Graphical abstract

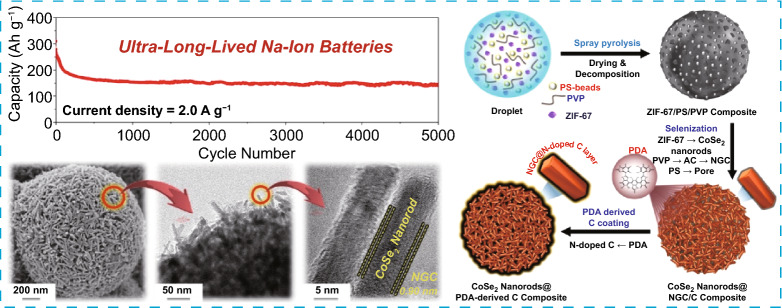

## Introduction

Recently, Na-ion batteries (NIBs) have drawn increasing attention as a feasible alternative to commercial Li-ion batteries (LIBs) in the energy storage field because Na has a rechargeable electrochemical reaction similar to Li of LIBs and is more widespread and abundant than lithium [1–6]. Accordingly, transitional metal selenides (TMSs) as promising anodes for NIBs have also been intensively investigated in recent times [1–8]. In general, TMSs show superior Na-ion storage capability than other transitional metal oxides and sulfides [3, 9]. Furthermore, TMSs have increased bond lengths and correspondingly weaker binding energies, which reduces the energy consumption of the sodiation/desodiation reaction [9, 10]. However, the practical applications of TMSs for NIBs are hampered by a large volume variation during cycling, which causes mechanical fracture and electrical contact loss, leading to poor cell cyclability [3, 5, 6, 11]. In particular, the capacity decay of cells for NIBs with TMS anodes is seriously induced under the carbonate-based electrolyte system, which has high activation-energy barrier for Na-ion diffusion on the structure and low stability during repeated cycles [12, 13]. Moreover, the low electrical conductivity of TMSs instigates high cell resistance, resulting in low specific capacities of cell [4–6]. Thus, to solve the problems of TMSs, particularly for poor cycle stability of NIBs under practically profitable carbonate-based electrolyte system, the structural design of TMSs electrode materials is essential and has been proved to be an effective strategy over the past decade [4, 5].

Metal–organic frameworks (MOFs) consisting of metal ion nodes and organic linkers have received much attention as precursors to prepare nanostructured TMSs/carbon composite with a high surface area and numerous pores [12, 14, 15]. Additionally, MOFs are a new class of porous material that offer advantages, such as adjustable pore sizes and controllable architectures [15, 16]. Because of these unique characteristics of MOFs, many studies employing MOFs as a precursor have been suggested as anode materials for NIBs recently [17–19]. As one of the MOFs, zeolitic imidazolate framework-67 (ZIF-67) composed of Co ions as metallic nodes and 2-methylimidazole (2-mIM) as an organic linker has also gained recognition as electrode materials [17, 20, 21]. Jiang et al. prepared nitrogen-doped carbon-coated Co_3_S_4_ nanocrystals through continuous carbonization and sulfidation processes using ZIF-67 as a precursor. The composite showed ultrafine nanocrystals with a diameter of approximately 5 nm. When utilized as the anode for NIBs, the composite demonstrated a specific capacity of 421 mAh g^−1^ at a current density of 0.1 A g^−1^ after 100 cycles [20]. Zhao et al. also prepared an architecture based on CoSe_2_ nanoparticles embedded in a carbon matrix with a conformal TiO_2_ film by coating TiO_2_ on ZIF-67 via sol-gel process. The as-prepared composite particles had a reversible Na storage capacity of 520 mAh g^−1^ at 0.1 A g^−1^, with a capacity retention of 78% after 200 cycles [21]. Even though most studies using ZIF-67 as precursors for enhanced electrode materials have been carried out, but the intrinsic low electrical conductivity of the TMS/carbon derived from MOFs and their poor structure integrity during repeated cycles are the major problem, which limited their practical application as anodes for NIBs with long cycle life [22, 23].

Hence, in this study, we introduce a synthetic strategy using the spray pyrolysis process to solve these limitations. Spray pyrolysis is an aerosol-assisted process and has recently gained prominence because it is a facile, cost-effective, and continuous process and thus is commercially available [24, 25]. The highly conductive and porous microspheres uniformly comprised of one-dimensional (1D) CoSe_2_ nanorods as building blocks, which are double-coated with ZIF-67-templated nitrogen-doped graphitic carbon (NGC) and polydopamine-derived carbon (PDA-derived C) were developed for the first time using a scalable spray pyrolysis process. The NGC layer surrounding the 1D CoSe_2_ nanorods are anticipated to act as primary transport pathways for direct electron transfer from the active materials, thereby supporting the rapid redox processes during the electrochemical process. Moreover, the additional double-coated PDA-derived N-doped C acts as secondary transport pathways to further support the electron transfer to the copper substrate and primary conductive pathways formed by NGC, along with the reinforcement to strengthen the anode structural integrity. Additionally, we introduce the porous channels in the TMS/C composite, allowing efficient wetting of the electrode and thereby promoting fast redox processes during the electrochemical testing. The synergetic effects of the rational synthesis approach, which enables dual C coating and porous channel, can be beneficial to overcome the intrinsic limitation of the MOF structure to achieve the anodes with extraordinary electrochemical performance for NIBs under practically profitable carbonate-based electrolyte system, particularly the ultra-long cycle life stability.

In this study, the formation mechanisms of the unique porous TMS/C composite microspheres using spray pyrolysis were comprehensively investigated by tracing the morphological and phasic features of the structure. We believe that the structural advancements presented in this study will benefit the research community in visualizing highly conductive and porous nanostructures for long-lived electrodes in various energy storage applications.

## Experimental

### Sample Preparation

#### Preparation of ZIF-67 Polyhedrons

The ZIF-67 polyhedrons were prepared by a facile solution-based precipitation method. Briefly, 50 mL aqueous solution of 0.05 M Co(NO_3_)_2_∙6H_2_O (Junsei Chemical Co., Ltd., 98%, Mw: 291.02) was added to 50 mL of another aqueous solution containing 0.4 M 2-methylimidazole (Acros Organics, 99%, M_w_: 82.10) and 6 mL of triethylamine (Alfa Aesar, 99%, M_w_: 101.19) to form a purple colloidal solution. The resultant suspension was stirred for 1 h to obtain a homogeneous dispersion. Subsequently, the solution was left undisturbed at ambient conditions for 24 h and allowed to settle down. The resulting purple precipitate was collected by centrifugation at 9000 rpm and washed several times with distilled water and ethanol. Finally, the as-prepared ZIF-67 polyhedrons were obtained by drying the sample overnight in a vacuum oven at 100 °C.

#### ***Preparation of P-CoSe***_***2***_***@NGC NR Microspheres***

Hierarchically porous P-CoSe_2_@NGC NR microspheres were prepared using a scalable spray pyrolysis technique. Firstly, 1.5 g of PVP (Daejung Chemicals and Metals Co., Ltd., Mw: 40,000) was dissolved in 100 mL of distilled water by stirring for 1 h. Subsequently, 3.0 g of ZIF-67 polyhedrons and 50 mL of PS nanobeads (*ϕ* = 40 nm, concentration = 0.1 g mL^−1^) suspension were added to the obtained solution and vigorously stirred for 2 h to prepare a final spray solution. The preparation method of PS nanobeads suspension has been described in our previous report [26]. The ZIF-67/PS/PVP spray solution was then transferred to an ultrasonic nebulizer connected to a vertical quartz reactor. The droplets generated by the nebulizer were fed through a quartz reactor fixed at 400 °C in an N_2_ atmosphere (10 L min^−1^). The schematic representation of the spray pyrolysis arrangement is illustrated in Scheme S1. The ZIF67/PS/PVP composite microspheres obtained after the spray pyrolysis process were transferred to a crucible together with a small alumina bottle containing Se powder and selenized for 4 h at 350 °C under Ar/H_2_ (vol = 95:5%) atmosphere to obtain P-CoSe_2_@NGC NR microspheres. The weight ratio of Se powder and the sample of ZIF67/PS/PVP was maintained at 3:1.

#### ***Preparation of P-CoSe***_***2***_***@PDA-C NR Microspheres***

The as-prepared P-CoSe_2_@NGC NR microspheres (0.1 g) were dispersed in 100 mL of tris buffer solution (0.01 M, pH: 8.7) to prepare the PDA-derived C-coated P-CoSe_2_@PDA-C NR microspheres. Subsequently, dopamine hydrochloride (Alfa Aesar, 99%, Mw: 189.64, 0.01 g) was added to the above solution and stirred for 12 h under ambient conditions. The PDA-coated P-CoSe_2_@NGC NR microspheres were then washed several times with distilled water, collected by centrifugation, and subsequently dried in an oven at 100 °C. Finally, P-CoSe_2_@PDA-C NR microspheres were obtained by heat treatment for 3 h at 400 °C in N_2_ atmosphere.

#### ***Preparation of Bare CoSe***_***2***_*** Hollow Microspheres***

For better comparison, bare CoSe_2_ hollow microspheres (without PVP and PS nanobeads) were also prepared through an identical spray pyrolysis and subsequent heat treatment processes. The spray solution was obtained by adding 0.15 mol of Co (NO_3_)_2_·6H_2_O to 300 mL of distilled water and stirring for 1 h. The temperature of the thermal reactor was maintained at 400 °C, and the flow rate of the carrier gas (air) was 10 L min^−1^. The as-prepared powder obtained by spray pyrolysis was selenized using Se powder at 400 °C for 6 h under Ar/H_2_ (vol = 95:5%) atmosphere to obtain bare CoSe_2_ hollow microspheres.

### Characterization Techniques

The morphology of the samples was examined through FE-SEM (Ultra Plus, Zeiss) and FE-TEM (JEM-2100F, JEOL). The phase and crystal structure of the samples were analyzed by XRD (D8 Discover with GADDS, Bruker) using Cu Κα radiation (*I* = 1.5418 Å). The chemical environment of elements in the samples was determined by XPS (K-Alpha, Thermo Fisher Scientific) using a monochromatic Al Κα radiation at 12 kV and 20 mA. The crystallinity of the carbonaceous materials in the prepared samples was investigated through Raman spectroscopy (Horiba Jobin–Yvon, HR800, LabRam). The surface area of the samples was measured by the BET method, where N_2_ was used as the adsorbate gas. The carbon content was quantified by TGA (Pyris 1, PerkinElmer) in an air atmosphere from 30 to 600 °C at a ramp rate of 10 °C min^−1^. The inductively coupled plasma-optical emission spectrometer (ICP-OES) analysis was performed to determine the elemental composition using Perkin Elmer OPTIMA 7300 DV. Elemental analysis (EA) technique based on high temperature (1200 ~ 1400 °C) combustion method was employed for quantitative analysis of C and N elements in the prepared samples (vario MICRO cube, Elementar). The amount of sample used for the analysis was 2.5 mg.

### Electrochemical Measurements

The electrochemical properties of the samples were measured using 2032-type coin cells. The working electrodes composed of active material, conductive carbon (Super-P), and sodium carboxymethyl cellulose as a binder in a mass ratio of 7:2:1 was prepared using a slurry casting method on a copper foil, which were subsequently dried overnight at 60 °C in a hot air oven. The circular electrodes (*ϕ* = 14 mm) with a mass loading of ~ 1.0 mg cm^−2^ (active material mass = 1.54 mg) were punched and transferred inside the glovebox. The Na metal and microporous polypropylene film were used as the counter electrode and separator, respectively. The electrolyte used was 1.0 M NaClO_4_ in a mixture of ethylene carbonate and dimethyl carbonate with a volume ratio of 1:1 with 5 wt% fluoroethylene carbonates. The coin cell was assembled at room temperature (22–28 °C) in an Ar-filled glove box. The electrochemical performances of the samples were evaluated using CV, charge–discharge testing, and EIS. The voltage window throughout the electrochemical tests was fixed at 0.001–3 V. The CV measurements of the samples were performed at a scan rate of 0.1 mV s^−1^. The charge–discharge testing of the samples was conducted at various current densities from 0.1 to 5.0 A g^−1^. The EIS data of the samples were collected in the frequency range of 100 kHz–0.01 Hz using the signal amplitude of 10 mV.

## Results and Discussion

### Physical Characterization Results

**Scheme 1:**
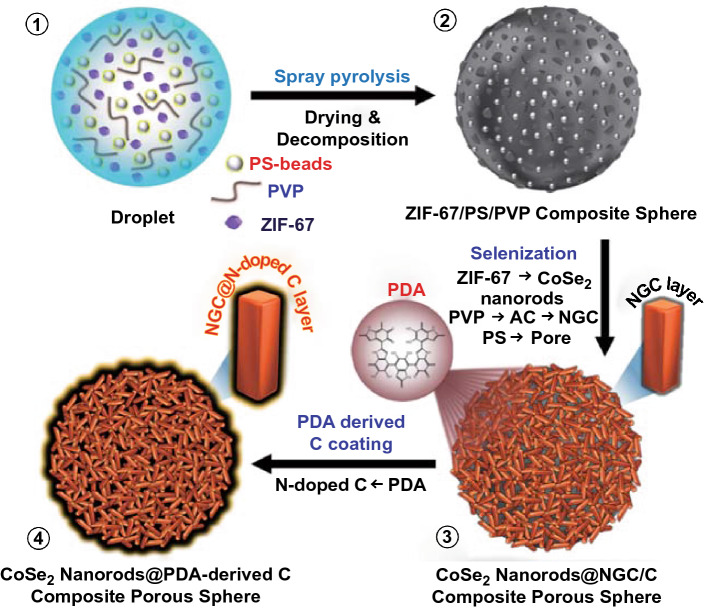
Schematic representation of formation mechanism (①–④) of the CoSe_2_ nanorods@PDA-derived C composite porous microspheres

The spray pyrolysis and subsequent multi-steps prepared three-dimensional (3D) porous microspheres containing ZIF-67-templated NGC and PDA-derived C-coated randomly oriented 1D CoSe_2_ nanorods. A detailed synthesis mechanism for the unique porous microspheres is systematically illustrated in Scheme 1. The ultrasonic nebulizer generated aqueous droplets uniformly composed of ZIF-67 polyhedrons, polyvinylpyrrolidone (PVP), and polystyrene (PS) nanobeads (*ϕ* = 40 nm, Fig. S1) as a Co precursor, carbon source, and pore-forming agent, respectively (Scheme 1-①). Before this, the ZIF-67 polyhedrons were synthesized via a facile solution-based method using 2-mIM and Co salt as starting materials with triethylamine (TEA) as an additive. The TEA amount was adjusted during the synthesis process to optimize the size of ZIF-67 polyhedrons (Fig. S2). ZIF-67 polyhedrons obtained without TEA form a flat-leaf structure due to the two-dimensional (2D) networking between the Co^2+^ ions and 2-mIM organic linker (Fig. S2a) [27]. However, an increase in the pH owing to the addition of TEA induce the acceleration of the nucleation rate of crystal, resulting in a decrease in the crystal size (Fig. S2b-c) [28]. Therefore, in this study, the ZIF-67 with a size of 70 nm obtained with 6 mL TEA was selected to be composited effectively in the microsphere structure during spray pyrolysis. The droplets generated by the nebulizer were dried and pyrolyzed through a vertical quartz reactor tube maintained at 400 °C, forming ZIF-67/PS/PVP composite microspheres (Scheme 1-②). Thermogravimetric analysis (TGA) was performed in an N_2_ atmosphere to confirm the thermal stability of PVP and PS nanobeads within the microspheres (Fig. S3). The TG curve (Fig. S3a) suggested that PVP remained thermally stable until 400 °C and then decomposed rapidly to form amorphous carbon (AC). Similarly, the TG curve (Fig. S3b) of PS implies complete decomposition to gaseous products at approximately 400 °C. However, it should be noted that the PS nanobeads remain inside the microspheres (as discussed later) during the spray pyrolysis due to the short residence time of droplets inside the quartz reactor (~ 6.3 s). Furthermore, the as-sprayed ZIF-67/PS/PVP composite precursor microspheres were selenized using Se powder at 350 °C for 4 h in the Ar/H_2_ (vol = 95:5%) atmosphere. During the selenization, ZIF-67 acted as a source for metallic-Co nuclei, which reacted with the H_2_Se gas formed due to the combination of Ar/H_2_ with Se to produce CoSe_2_ crystals nuclei. Due to high surface energy, the CoSe_2_ nuclei aggregated rapidly and lower down their surface energy via intrinsic crystal orientation [12, 29]. Finally, this directional crystallization resulted in the growth of CoSe_2_ crystals nuclei into randomly oriented 1D CoSe_2_ nanorods as building blocks (Scheme 1-③). Moreover, the nitrogen-rich organic ligands in the ZIF-67 polyhedrons converted to NGC well coated surrounding the CoSe_2_ nanorods. Similarly, the nitrogen-rich species in PVP were first carbonized to AC and subsequently transformed to NGC due to the catalytic effect of the Co nuclei present in ZIF-67 polyhedrons. The presence of an NGC layer acted as a primary conductive path for a rapid electron transfer. Moreover, the complete decomposition of PS nanobeads to gaseous products left numerous pores in the composite, which facilitated the homogeneous formation of non-agglomerated CoSe_2_ nanorods over the nanostructure through effective penetration of H_2_Se gas and guaranteed the enough space between nanorods to prevent agglomeration during the selenization process. The detailed explanation for explicit role of PS nanobeads is included in the supporting information file along with the relevant figure (Fig. S4). The entire selenization process produced 3D porous microspheres well supported by the MOF-templated NGC-coated 1D CoSe_2_ nanorods (denoted as P-CoSe_2_@NGC NRs) (Scheme 1-③). In the final step, the PDA-derived C layer as a secondary conductive path was coated onto the P-CoSe_2_@NGC NRs composite microspheres via the facile solution-based coating process. Further, the PDA-derived C-treated composite microspheres were carbonized at 400 °C in the N_2_ atmosphere for 3 h (Scheme 1-④). The overall synthesis process generated highly conductive 3D porous microspheres containing NGC-coated 1D CoSe_2_ nanorods surrounded by a highly conductive PDA-derived C layer as a secondary transport path for the fast charge transfer (denoted as P-CoSe_2_@PDA-C NR). The double-coated C layer increased the integrity of the prepared nanostructure and served as primary and secondary transport pathways for rapid electron transfer that supported the exceptional electrochemical performance such as the ultra-long cycling stability.

**Fig. 1:**
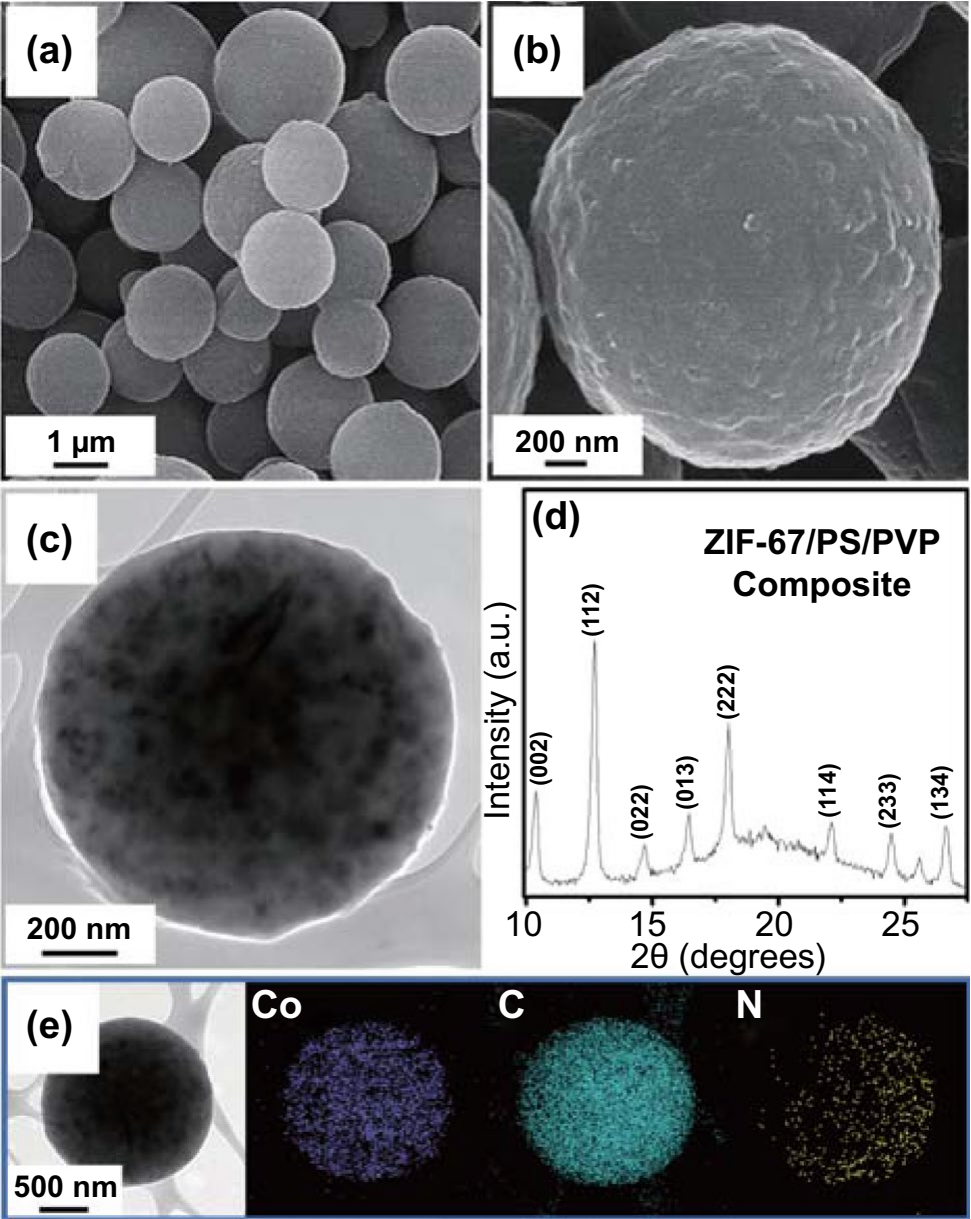
**a**, **b** FE-SEM images, **c** TEM image, **d** XRD pattern, and **e** elemental mapping images of the ZIF-67/PS/PVP composite microspheres obtained after spray pyrolysis at 400 °C

A comprehensive morphological and crystal structure analysis of the prepared nanostructures was performed after each step of the synthesis to elucidate the formation mechanism. Figure 1 shows the microstructural and phase analysis results of the as-prepared ZIF-67/PVP/PS composite microspheres obtained after spray pyrolysis. The field emission scanning electron microscopy (FE-SEM) image (Fig. 1a) illustrates the formation of well-dispersed and non-aggregated microspheres with an average diameter of 1.5 µm. In addition, a slightly rough surface of the microsphere was evident (Fig. 1b) due to the ZIF-67 polyhedrons and PS nanobeads within the structure. The TEM image in Fig. 1c firmly supports the presence of ZIF-67 particles (black spots) with polyhedron-type morphology in the microsphere. The X-ray diffraction (XRD) pattern of the as-sprayed composite microspheres (Fig. 1d) indicated well-resolved sharp diffraction peaks primarily due to the diffraction peaks of ZIF-67 polyhedrons within the structure, which is also confirmed in Fig. S2d. However, a close examination showed a broad diffraction peak at 2*θ* = 19°, suggesting that the as-sprayed composites were in amorphous-like phase. The amorphous nature of the composite microspheres was attributed to the thermal decomposition of PVP in the structure to AC. The elemental mapping images in Fig. 1e suggest that Co, C, and N from ZIF-67 polyhedrons and PVP constitutes the microspheres.

**Fig. 2:**
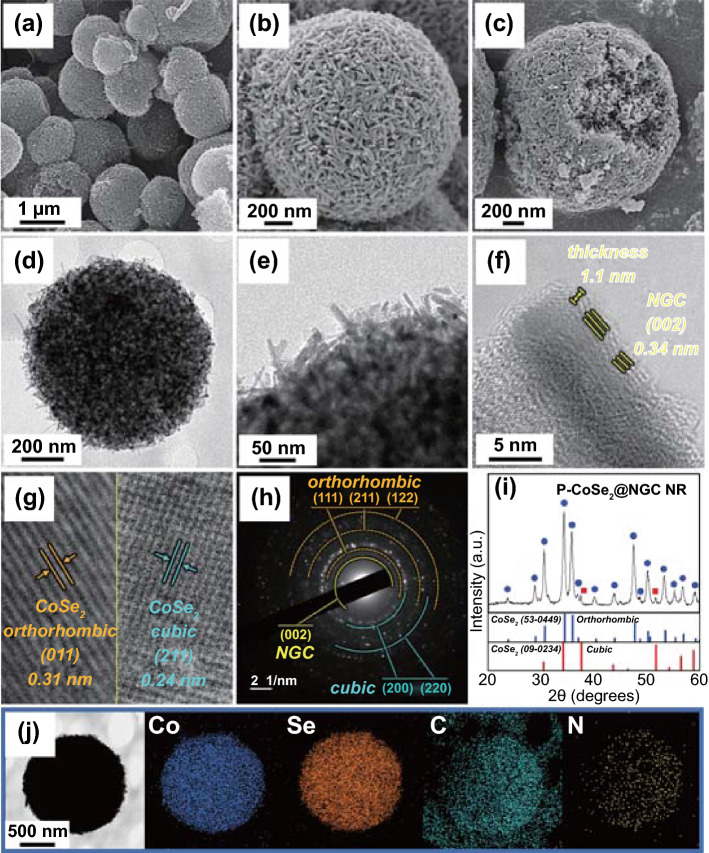
Morphologies, SAED, XRD patterns, and elemental mapping images of the P-CoSe_2_ @NGC NR microspheres by spray pyrolysis and subsequent selenization at 350 °C: **a, b** FE-SEM images, **c** fractured sphere’s FE-SEM image, **d-f** TEM images, **g** HR-TEM image, **h** SAED pattern, **i** XRD pattern, and **j** elemental mapping images

The 3D porous microspheres comprising ZIF-67 templated NGC-coated randomly oriented 1D CoSe_2_ nanorods as building blocks (denoted as P-CoSe_2_@NGC NRs) were obtained after the selenization process of the as-sprayed ZIF-67/PS/PVP composite microspheres at 350 °C for 4 h under Ar/H_2_ (vol = 95:5%) gas mixture. Selenium powder was used as a precursor to combine with H_2_ to form H_2_Se gas. It should be noted that the pores generated due to the decomposition of homogeneously distributed PS nanobeads in the composite structure regulates the efficient penetration of the H_2_Se gas and prevents the aggregation of adjacent CoSe_2_ nanorods. The composite microspheres without PS nanobeads did not form the uniform nanorods-type morphology of CoSe_2_ due to insufficient H_2_Se gas penetration inside of the structure (Fig. S5 and the corresponding discussion). Figure 2 illustrates the resulting nanostructure morphology of P-CoSe_2_@NGC NR after the selenization process. The low-magnification FE-SEM image (Fig. 2a) revealed that the obtained P-CoSe_2_@NGC NR microspheres maintained their spherical shape (mean diameter of approximately 1.0 µm) with a uniform distribution of randomly oriented rod-shaped nanocrystals throughout the external and internal structures (Fig. 2b, c). Moreover, the heating process resulted in the complete thermal decomposition of various organic ligands constituting the ZIF-67 polyhedrons, PS nanobeads, and PVP, thereby shrinking the microspheres. The TEM image (Fig. 2d, e) demonstrated the formation of spherical microspheres with numerous pores in the structure. The randomly oriented 1D nanorods that supported uniformly throughout the internal and external structure was also evident (Fig. 2e). In addition, the TEM image (Fig. 2f) showed that the rod-shaped nanocrystals (length = 32 nm and diameter = 7 nm) constituting the microsphere were surrounded by an NGC layer with a thickness of approximately 1.1 nm and well-resolved lattice fringe distance of 0.34 nm, which corresponds to the (002) plane of the GC. Several nitrogen-rich organic units in PVP and ZIF-67 polyhedrons acted as the nitrogen-source during the heat treatment process. Similarly, the AC produced from the carbonization of PVP converted to NGC due to the catalytic nature of the metallic-Co nuclei present in the ZIF-67. The NGC layer surrounding the 1D CoSe_2_ nanorods enhanced the overall electrical conductivity of the microspheres due to the high electronegativity of the nitrogen atom compared to that of the carbon atom [8, 30]. Moreover, the NGC-coated layer acted as a primary transport pathway for the rapid electron transfer, thereby supporting the rapid redox processes during the electrochemical process. The high-resolution TEM (HR-TEM) image in Fig. 2g suggests a bi-phasic crystal structure with well-resolved lattice fringes of separation of 0.31 and 0.24 nm, corresponding to the (011) and (211) crystal planes of the orthorhombic CoSe_2_ (o-CoSe_2_) and cubic CoSe_2_ (c-CoSe_2_), respectively. The selected area electron diffraction (SAED) pattern (Fig. 2h) firmly supported the above results with well-resolved diffraction rings corresponding to the bi-phasic crystal lattices of o-CoSe_2_ and c-CoSe_2_, along with the NGC phases. The XRD pattern in Fig. 2i also indicates sharp peaks primarily associated with the mixed phases of nanocrystalline o-CoSe_2_ and c-CoSe_2_ crystal structures and is consistent with the HR-TEM/SAED results. Notably, the diffraction peaks associated with o-CoSe_2_ were more prominent than the diffraction peaks associated with c-CoSe_2_. Additionally, the mean crystallite size of CoSe_2_ in P-CoSe_2_@NGC NR microspheres was 16 nm, which was calculated using the Scherrer formula by considering the highly intense (111) diffraction peak in the XRD pattern. The elemental mapping results in Fig. 2j show the homogeneous distribution of Co, Se, C, and N elements in the P-CoSe_2_@NGC NR microspheres, indicating the uniform composition of numerous CoSe_2_ nanorods with NGC. Moreover, ICP-OES analysis was carried out to quantitatively analyze the ratio of Co and Se elements in the P-CoSe_2_@NGC NR microspheres. The contents of Co and Se elements were 20.4 and 57.6 wt%, respectively, which corresponds to a molar ratio of 1: 2.1 (Table S1).

**Fig. 3:**
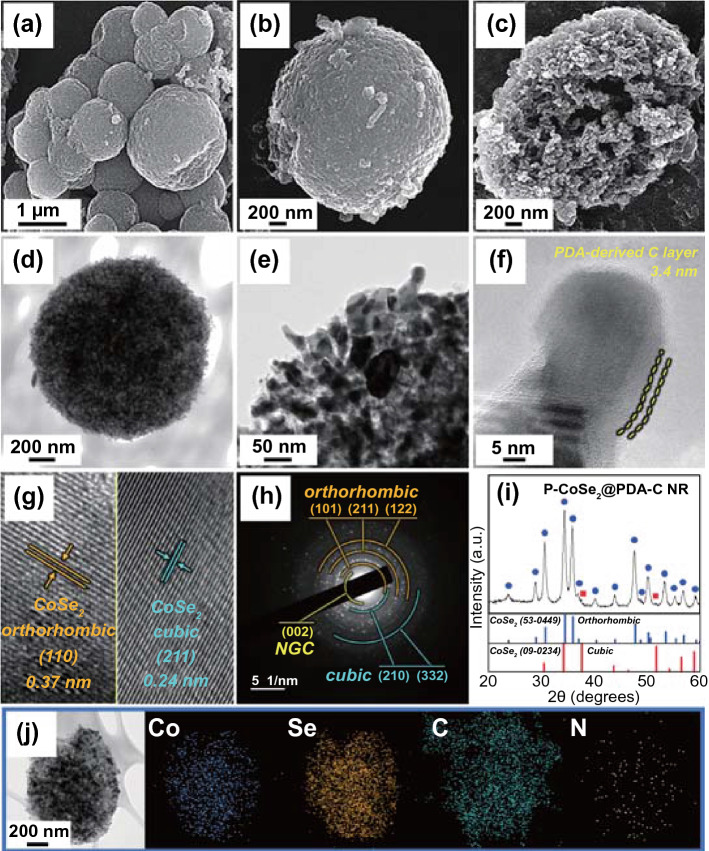
Morphologies, SAED, XRD patterns, and elemental mapping images of the P-CoSe_2_@PDA-C NR microspheres: **a**, **b** FE-SEM images, **c** fractured sphere’s FE-SEM image, **d**–**f** TEM images, **g** HR-TEM image, **h** SAED pattern, **i** XRD pattern, and **j** elemental mapping images

To achieve exceptional Na-ion storage performance of the cell with ultra-long cycling stability, the PDA-derived N-doped C was coated onto the P-CoSe_2_@NGC NRs precursor microspheres by a facile solution-based coating method and subsequent carbonization at 400 °C for 3 h in the N_2_ atmosphere. Figure 3 shows the resulting nanostructure, PDA-derived C-coated composite microspheres comprising ZIF-67 templated NGC-coated 1D CoSe_2_ nanorods (P-CoSe_2_@PDA-C NRs). The low-magnification FE-SEM image (Fig. 3a) shows the morphology of the P-CoSe_2_@PDA-C NR that is intact with an average diameter of approximately 1.0 μm. Further, the high-magnification FE-SEM images in Fig. 3b, c suggests that the entire coating and subsequent heat treatment process has marginal effect on the external and internal surfaces of the composite microspheres. In particular, the randomly oriented 1D CoSe_2_ nanorods becomes slightly shorter and broader. Additionally, the TEM images (Fig. 3d, e) confirmed the FE-SEM results with 3D porous microspheres supported by 1D CoSe_2_ nanorods and numerous pores throughout the structure as dark and bright regions, respectively. The high-magnification TEM image in Fig. 3f indicates that the coating process forms a uniform carbon layer with a coating thickness of *ca.* 3.4 nm surrounding the 1D rod-shaped nanocrystals. Furthermore, the HR-TEM image (Fig. 3g) suggested that the coating process did not alter the bi-phasic crystal structure of the composite microspheres with well-resolved lattice fringes of separation of 0.37 and 0.24 nm, corresponding to the (110) and (211) crystal planes of the o-CoSe_2_ and c-CoSe_2_ phases, respectively. The SAED pattern (Fig. 3h) also demonstrated well-resolved diffraction rings corresponding to the o-CoSe_2_, c-CoSe_2_, and NGC phases. These results further confirmed that the CoSe_2_ crystal phase was maintained even after the additional heat treatment, and a thin carbon coating layer from the PDA-derived carbon was uniformly coated along with the NGC. In particular, the PDA-derived C coating layer acted as secondary transport pathways to further support the fast electron transfer and primary conductive pathways formed by NGC. This dual carbon coating strategy envisages exceptional electrochemical properties due to the rapid redox process in addition to increasing the mechanical integrity of the prepared composite nanostructure. Furthermore, the XRD pattern in Fig. 3i is in good accordance with the above results, indicating the evident coexistence of sharp diffraction peaks mainly related to the nanocrystalline o-CoSe_2_ crystal structure with a small fraction of c-CoSe_2_ crystal phase. The average crystallite size of CoSe_2_ in P-CoSe_2_@PDA-C NR microspheres was 15 nm, which was calculated using the Scherrer formula from the highly intense (111) peak in the XRD pattern. The average size of the CoSe_2_ nanocrystals was similar even after the additional heat treatment, suggesting adequate suppression of the crystal growth by the carbon layer surrounding the CoSe_2_ crystallites. The elemental mapping results in Fig. 3j show the homogeneous dispersion of Co, Se, C, and N elements in the P-CoSe_2_@PDA-C NR composite microspheres. Overall, the above results suggest that the PDA-derived C and NGC were successfully coated onto the 1D CoSe_2_ nanorods, producing synergetic effects to support the kinetically favored redox processes and further enhance the overall electrochemical performance.

**Fig. 4:**
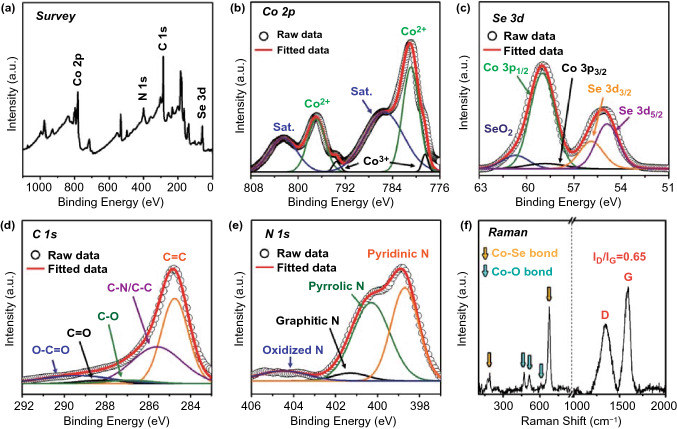
XPS survey spectrum, core-level XPS spectra, and Raman spectrum: **a** XPS survey spectrum, **b** Co 2*p*, **c** Se 3*d*, **d** C 1*s*, **e** N 1*s*, and **f** Raman spectrum of the P-CoSe_2_@PDA-C NR microspheres

The X-ray photoelectron spectroscopy (XPS) further determined the chemical and bonding environment of various elements in the P-CoSe_2_@PDA-C NR microspheres. Figure 4a shows the XPS survey spectrum, consisting of photoelectronic signals corresponding to Co 2*p*, Se 3*d*, C 1*s*, and N 1*s* [12, 31]. The Co 2*p* spectrum in Fig. 4b demonstrates well-defined peaks corresponding to the two spin-orbital doublets, Co 2*p*_3/2_ and Co 2*p*_1/2_, along with the two shake-up satellite peaks for Co (labeled “Sat”). The deconvoluted spectrum exhibited well-resolved peaks at binding energies of 780.9 eV (Co 2*p*_3/2_) and 796.9 eV (Co 2*p*_1/2_) assigned to the Co^2+^ species in the CoSe_2_ [12, 31]. The additional peaks at 778.5 eV and 793.5 eV for Co 2*p*_3/2_ and Co 2*p*_1/2_, respectively, were attributed to the partial surface oxidation of the composites in air [12]. Furthermore, the two shake-up satellite peaks for Co 2*p* signal corresponded to the antibonding orbitals between Se and Co atoms [12]. The deconvoluted Se 3*d* spectrum (Fig. 4c) demonstrated two well-fitted peaks for Se 3*d*_5/2_ (54.9 eV) and Se 3*d*_3/2_ (55.9 eV), which were attributed to the interaction of Se with metallic-Co in CoSe_2_ [32, 33]. Moreover, the peaks in the range of 58–60 eV corresponded to Co 3*p* on the surface of the composites [12]. The additional peak at 60.7 eV was attributed to the Se-O bond formed due to the surface oxidation of Se under atmospheric conditions [12]. The C 1*s* spectrum in Fig. 4d is deconvoluted into five distinct peaks at binding energies of 284.8, 285.6, 286.9, 288.2, and 289.4 eV, corresponding to the C–C(*sp*^2^), C–C/C–N, C–O, C=O, and C–O=O species, respectively [12, 30, 34]. The highest intensity of the C = C peak indicated the graphitic carbon formation during the selenization process [35]. In addition, the presence of C–N/C–C peaks suggested the presence of an N-doped C [8, 12]. The N doping improved the overall electrical conductivity due to the high electronegativity of the nitrogen atom compared to the carbon atom. The high-resolution N 1*s* spectrum in Fig. 4e showed four types of N species: pyridinic N (398.7 eV), pyrrolic N (400.3 eV), graphitic N (401.3 eV), and oxidized N (404.5 eV), confirming the N doping in the coated carbon [30, 36]. Further, Raman spectroscopy was employed to analyze the crystalline properties of the carbonaceous products in P-CoSe_2_@PDA-C NR microspheres (Fig. 4f). The established signatures of D- and G-bands were identified at 1344 and 1593 cm^−1^, respectively [31, 37, 38]. Moreover, the relative intensity ratio of the D- and G-band (*I*_D_*/I*_G_) measured the crystallinity of the carbonaceous material [37, 38]. The *I*_D_*/I*_G_ value of 0.65 for P-CoSe_2_@PDA-C NR microspheres suggested the highly crystalline nature of the carbon. However, the non-coated P-CoSe_2_@NGC NR microsphere exhibited a lower *I*_*D*_*/I*_*G*_ value of 0.46 (Fig. S6), suggesting that the introduction of the PDA-derived C layer increased the non-*sp*^3^ carbon content or disordered carbon in the nanostructure. The increase in the disorderedness was attributed to the molecular structure of PDA (Fig. S7). The result indicated that the carbon derived from PDA had abundant N element that introduced defects, thereby increasing the D-band and decreasing the crystallinity. These observations are in good agreement with the elemental analysis (EA) results obtained for the composite microspheres before and after the PDA treatment (Table S2). The N wt% for P-CoSe_2_@PDA-C NR and P-CoSe_2_@NGC NR microspheres were approximately 4 and 2 wt%, respectively, which firmly supported the above discussion. Consequently, an arrangement of the dual carbon coating composed of NGC and N-doped carbon resulted in an overall high electrical conductivity for P-CoSe_2_@PDA-C NR microspheres. Moreover, the Raman spectrum demonstrated well-resolved peaks at 192 and 676 cm^−1^, corresponding to the Co–Se bonds [31, 39]. Furthermore, the slightly lower intensity peaks assigned to the trace amounts of Co–O bonds generated by surface oxidation were observed at 471, 512, and 608 cm^−1^ [31, 40]. The N_2_ adsorption–desorption isotherms of P-CoSe_2_@PDA-C NR microspheres are obtained to validate the Raman results, as shown in Fig. S8a. The isotherms suggested a high volume of gas adsorption, which was primarily attributed to the micropores and mesopores present throughout the structure that subsequently resulted in a high Brunauer–Emmett–Teller (BET) surface area of 164 m^2^ g^−1^. The surface defects present in the PDA-derived carbon-coating layer formed the micropores and mainly induced by the N-species present in the structure. The mesopores were attributed to the thermal decomposition of the PS nanobeads (40 nm). Furthermore, the ZIF-67-derived carbon skeleton contributed to the sample mesoporosity. In contrast, the P-CoSe_2_@NGC NR microspheres before the PDA treatment showed a relatively low BET surface area of 34 m^2^ g^−1^ (Fig. S8b) mainly due to the absence of numerous surface defects, which was evident from the low volume of the adsorbed N_2_. These results are consistent with the Barrett-Joyner-Halenda pore-size distribution curve, indicating a slightly low average pore diameter (approximately 30 nm) for P-CoSe_2_@PDA-C NR (Fig. S8c) compared to that of the P-CoSe_2_@NGC NR (approximately 40 nm, Fig. S8d). The narrow peak at *ca.* 4 nm as attributed to the tensile-strength effect [41]. Furthermore, the carbon content in the P-CoSe_2_@PDA-C NR microspheres is quantified using the TG curve in Fig. S8e. The result suggested a sharp weight loss from 360 °C due to the concomitant conversion of CoSe_2_ to CoSeO_4_ and SeO_2_ and then to Co_3_O_4_, along with the decomposition of AC and carbon-coated layers derived from PDA and NGC to gaseous products [42]. Therefore, the estimated C content in the P-CoSe_2_@PDA-C NR microspheres using TG analysis was 33 wt% and consistent with the EA results (Table S2). Similarly, the C content in P-CoSe_2_@NGC NR microspheres obtained through the TG (Fig. S8f) and EA results was *ca.* 16 wt%. Overall, the above discussion suggests that the coating process forms a highly conductive coating layer that facilitates the enhanced diffusion of charge species along with the insertion of more Na-ions owing to the numerous defects induced by the N-species in the PDA-derived C [43, 44]. These synergetic effects consequently envisage superior electrochemical performance such as long-term cycling stability.

Bare CoSe_2_ hollow microspheres without PVP and PS nanobeads were prepared as one of the comparison samples to prove the structural advantages of the P-CoSe_2_@PDA-C NR microspheres. The morphology and crystal structure of the precursor powder obtained immediately after spray pyrolysis in an air atmosphere are shown in Fig. S9. The FE-SEM micrographs (Fig. S9a) exhibited the formation of irregular-sized hollow microspheres primarily due to the Ostwald ripening. The surface of the hollow microsphere (Fig. S9b, c) demonstrated highly agglomerated and diffused grains, which was evident due to the absence of carbon for restricting the crystal growth. The XRD pattern (Fig. S9d) indicated a distinct nanocrystalline Co_3_O_4_ phase without impurity. The obtained bare Co_3_O_4_ hollow microspheres further underwent selenization at 400 °C to form pristine CoSe_2_ hollow microspheres (Fig. S10). The FE-SEM micrograph in Fig. S10a reveals a spherical-shaped morphology with an average diameter of 1.0 μm. The surface of hollow microspheres comprises closely packed large-sized grains, as evident from Fig. S10b, c. These grains were considered CoSe_2_ crystals that were formed during the selenization process, which became uncontrollable due to the absence of carbon for restricting the crystal growth. The TGA curve in Fig. S10d indicates negligible traces of carbon in the bare CoSe_2_ hollow microspheres. The XRD pattern (Fig. S10e) pattern further proved the total phase conversion from the Co_3_O_4_ crystal phase to the c-CoSe_2_ phase during the selenization process. Additionally, a large crystallite size of *ca.* 41.3 nm for pristine CoSe_2_ hollow microspheres using the Scherrer formula further validated the FE-SEM results. Moreover, the low BET surface area (0.86 m^2^ g^−1^; Fig. S10f) and the pore size distribution curve (Fig. S10g) suggests the complete absence of the pores for the bare CoSe_2_ hollow microspheres.

### Electrochemical Results

**Fig. 5:**
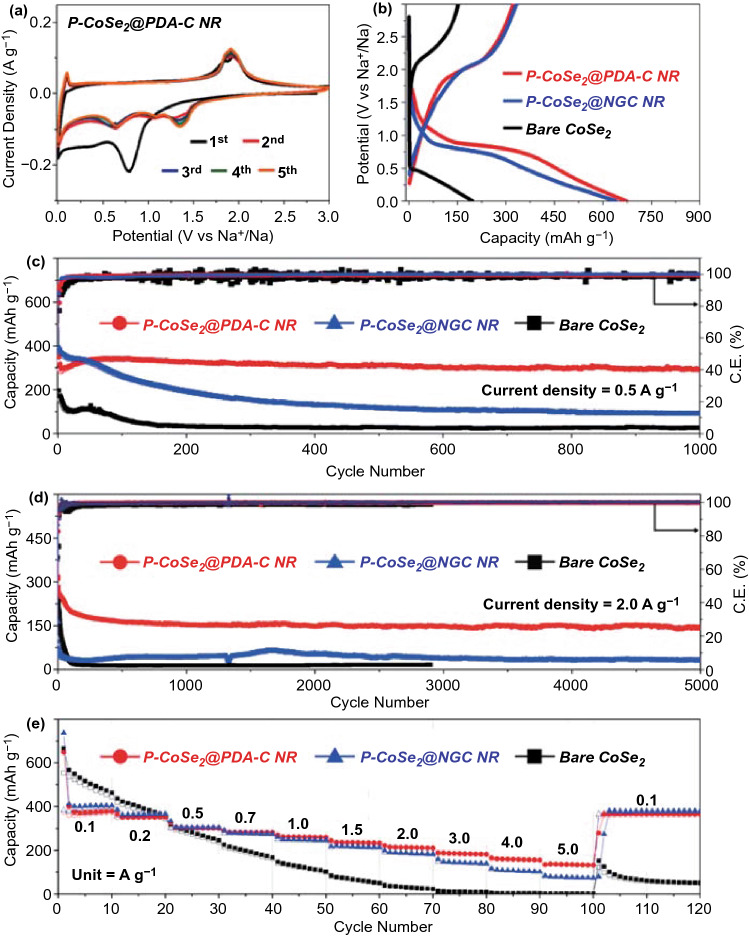
Electrochemical properties of the P-CoSe_2_@PDA-C NR, P-CoSe_2_ @NGC NR, bare CoSe_2_ hollow microspheres: **a** CV curves of the P-CoSe_2_@PDA-C NR microspheres, **b** initial discharge/charge curves at a constant current density of 0.5 A g^−1^, **c** cycle performances at current density of 0.5 A g^−1^, **d** cycle performances at current density of 2.0 A g^−1^, and **e** rate performances

The as-prepared well-designed P-CoSe_2_@PDA-C NR microspheres were further subjected to the Na-ion storage performance and compared with P-CoSe_2_@NGC NR and bare CoSe_2_ hollow microspheres to validate the structural advantages, as shown in Fig. 5. A carbonate-based electrolyte was used in this study although it could react with an anionic group, leading to poor cycle stability of the cell in NIBs. Nevertheless, the carbonate-based electrolyte system has low cost, simple preparation, low toxicity, environmental friendliness, and operates for a wide window range (0.001–3.0 V). The system exhibits a high-capacity utilization compared to the ether-based system, thereby making NIBs more profitable commercially [45, 46]. The cyclic voltammetry (CV) results for P-CoSe_2_@PDA-C NR microspheres over the first five cycles at a scan rate of 0.1 mV s^−1^ in the voltage window of 0.001–3.0 V are presented in Fig. 5a. In the first cathodic scan, the weak and broad region located at 1.16 V corresponded to the formation of Na_*x*_CoSe_2_ as an intermediate with the insertion of Na-ions into CoSe_2_ [47, 48]. Additionally, the sharp and weak broad peak at 0.79 and 0.38 V indicated the conversion reaction of Na_*x*_CoSe_2_ to metallic-Co and Na_2_Se accompanied by the decomposition of the electrolyte and formation of a stable solid-electrolyte interphase (SEI) film [47–49]. Furthermore, the sharp cathodic peak centered at 0.001 V suggested the insertion of Na-ions into the carbon [50, 51]. During the first anodic scan, peaks at 0.10, 1.84, and 1.95 V suggest desodiation from carbon, formation of Na_*x*_CoSe_2_ intermediate from metallic-Co and Na_2_Se, and CoSe_2_ and Na-ion formation from Na_*x*_CoSe_2_ intermediates, respectively [13, 31, 52]. However, the slightly different redox processes from the second cycle were observed due to the formation of ultrafine CoSe_2_ crystals, which is a well-known phenomenon in transition metal chalcogenide anode materials [7, 53–55]. For instance, three reduction peaks at 1.35, 1.09, and 0.64 V were surfaced, while the two oxidation peaks previously located at 1.84 and 1.95 V merged into a single peak centered at 1.91 V [47, 48]. In contrast, the peak initially centered at 0.10 V remained intact. The first reduction peak at 1.35 V corresponded to the intercalation of Na-ions, whereas the two reduction peaks at 1.09 and 0.64 V represented the two conversion reactions that formed CoSe/Na_2_Se and Co/Na_2_Se, respectively [47–49]. The single oxidation peak centered at 1.91 V corresponds to the formation of CoSe_2_ from metallic-Co and Na_2_Se as the reaction kinetics is improved by the formation of ultrafine crystal that resulted in instant formation and desodiation of the Na_*x*_CoSe_2_ intermediate form [47, 56, 57]. From the second scan, the exactly overlapping CV profiles indicated highly reversible redox reactions inside the cell. The CV curves of P-CoSe_2_@NGC NR in Fig. S11a show similar CV characteristics with few exceptions, mainly during the first cathodic process. For instance, the cell exhibited a high-intensity cathodic peak at a lower voltage side (0.50 V), which was accompanied by a shoulder peak at 0.72 V, although the involved redox reactions were similar to those observed in the P-CoSe_2_@PDA-C NR microspheres. A slight shift in the voltage could be attributed to the difference in the conductivity of the two samples that consequently affected the involved redox processes. However, with the second CV cycle, the cathodic/anodic profiles analogous to P-CoSe_2_@PDA-C NR microspheres implied similarly involved reaction kinetics. In contrast, the bare CoSe_2_ hollow microspheres in Fig. S11b demonstrate high-intensity cathodic/anodic peaks primarily caused by the crystalline nature of the prepared sample. In addition, the low voltage hysteresis between the cathodic and anodic scan suggested that the kinetics involved were mainly diffusion-controlled compared to P-CoSe_2_@PDA-C and P-CoSe_2_@NGC NR, where the redox processes were primarily surface or capacitive-controlled. The complete reaction mechanism involved during the discharge and charge processes is summarized below:

For the discharge process:

1 $${\mathrm{CoSe}}_{2}+x{\mathrm{Na}}^{+}+x{\mathrm{e}}^{-}\to {\mathrm{Na}}_{x}{\mathrm{CoSe}}_{2}$$

2 $${\mathrm{Na}}_{x}{\mathrm{CoSe}}_{2}+\left(2-x\right){\mathrm{Na}}^{+}+(2-x){\mathrm{e}}^{-}\to \mathrm{CoSe}+{\mathrm{Na}}_{2}\mathrm{Se}$$

3 $$\mathrm{CoSe}+2{\mathrm{Na}}^{+}+2{\mathrm{e}}^{-}\to \mathrm{Co}+{\mathrm{Na}}_{2}\mathrm{Se}$$

For the charge process:

4 $$\mathrm{Co}+2{\mathrm{Na}}_{2}\mathrm{Se}\to {\mathrm{CoSe}}_{2}+4{\mathrm{Na}}^{+}+4{\mathrm{e}}^{-}$$

The assembled cells were subjected to the galvanostatic discharge/charge process for structural merits evaluation. The initial discharge/charge profiles of the as-prepared composite microspheres at a current density of 0.5 A g^−1^ are shown in Fig. 5b. The obtained potential profiles were consistent with the CV results, with well-resolved discharge and charge plateaus at 0.83 and 1.98 V, respectively. The bare CoSe_2_ hollow microspheres exhibited an indistinct discharge/charge profile due to the extreme polarization (the voltage difference, that is, Δ*V* between the charge and discharge voltage profile, which primarily arose due to the slow diffusion of the charge species during the redox processes) at a high current density of 0.5 A g^−1^. Therefore, the initial discharge/charge profile of the bare CoSe_2_ hollow microspheres was also obtained at a low current density of 0.1 A g^−1^ to obtain more accurate discharge and charge plateaus (Fig. S12). Furthermore, the initial discharge capacities of the P-CoSe_2_@PDA-C NR, P-CoSe_2_@NGC NR, and bare CoSe_2_ hollow microspheres were 673, 640, and 197 mAh g^−1^, respectively, with a corresponding Coulombic efficiency (CE) value of 48%, 52%, and 77%, respectively. The slightly lower CE for P-CoSe_2_@PDA-C NR was attributed to the higher carbon content than other structures (Table S2), resulting high initial irreversible capacity loss. The magnified discharge/charge profiles for all composite microspheres in Fig. S13 suggests that the P-CoSe_2_@PDA-C NR exhibits the lowest polarization potential (Δ*V* = 1.14 V) than P-CoSe_2_@NGC NR (Δ*V* = 1.27 V) and bare CoSe_2_ hollow microspheres (Δ*V* = 1.98 V), implying the fast redox kinetics primarily due to the improved electronic and ionic conductivity of P-CoSe_2_@PDA-C NR. The synergetic effects of the dual coating with NGC and PDA-derived C coating as primary transport and secondary conductive pathways, respectively, improved the overall electronic/ionic conductivities and ensured a rapid charge transfer during the redox processes. Moreover, the numerous pores confirmed the superior electrolyte penetration that reduced the effective charge transport lengths by allowing fast diffusion of the charge species in the P-CoSe_2_@PDA-C NR microspheres.

The prepared composite microspheres are tested for their cycling stability performance at current densities of 0.5 and 2.0 A g^−1^, as shown in Fig. 5c, d. The as-prepared P-CoSe_2_@PDA-C NR demonstrated superior cycling stability performance over 1000 cycles at 0.5 A g^−1^ than the P-CoSe_2_@NGC NR and bare CoSe_2_ hollow microspheres (Fig. 5c). The P-CoSe_2_@PDA-C NR exhibited an initial discharge capacity of 673 mAh g^−1^ with a CE of 48% compared to 640 and 197 mAh g^−1^ of P-CoSe_2_@NGC NR and bare CoSe_2_ hollow microspheres, respectively. In addition, the cells featuring P-CoSe_2_@NGC NR and bare CoSe_2_ hollow microspheres exhibited an initial CE of 52% and 77%, respectively. During the few initial cycles, P-CoSe_2_@PDA-C NRs demonstrated an increase in the capacity mainly due to the formation of polymeric gel-like films on the microsphere surface, as reported previously [35, 58, 59]. However, as cycling proceeded, the P-CoSe_2_@PDA-C NR had much stable discharge capacities even after prolonged cycling than the P-CoSe_2_@NGC NR and bare CoSe_2_ hollow microspheres, which exhibited constant capacity fading. For instance, after 1000 consecutive discharge/charge cycles, the P-CoSe_2_@PDA-C NR had a discharge capacity of 291 mAh g^−1^ with an average capacity decay rate of only 0.017% (considered after the second cycle). In contrast, the P-CoSe_2_@NGC NR and bare CoSe_2_ hollow microspheres had a discharge capacity of 90 and 26 mAh g^−1^, respectively, at the end of the 1000th cycle, with much higher average capacity decay rate of 0.077% and 0.085%. Similar capacity trends are observed for the prepared composite microspheres when cycled at a higher current density of 2.0 A g^−1^ for ultra-long cycling life (5000 cycles) in Fig. 5d. As observed, the P-CoSe_2_@PDA-C NR microspheres exhibits initial capacity decrease till 150 cycles which was mainly induced by the transformation of the crystalline structure to a stable amorphous-like structure during the cycling [60, 61]. The P-CoSe_2_@PDA-C NR composite had a highly stable discharge capacity of 142 mAh g^−1^ at the end of the 5000th cycle (average capacity decay rate of 0.011% only). However, the P-CoSe_2_@NGC NR and bare CoSe_2_ hollow microspheres exhibited poor capacity retention during the initial 70 cycles. In particular, the bare CoSe_2_ exhibits high initial CE (~ 74%) compared to the P-CoSe_2_@NGC NR (~ 35%), which was due to its high crystalline nature with almost no carbon content. As a result, a sharp capacity decrease of P-CoSe_2_@NGC NR compared to bare CoSe_2_ was observed during the initial several cycling. However, the non-porous and non-conductive structure of bare CoSe_2_ consequently resulted in poor capacity retention and inferior cycling performance. These observations are more pronounced from the fact that the capacity retention reduced to 31 mAh g^−1^ at the end of the 5000th cycle for the P-CoSe_2_@NGC NR composite microspheres, whereas the cell featuring bare CoSe_2_ hollow microspheres stopped working after 2900 cycles. The overwhelming cycling stability of the P-CoSe_2_@PDA-C NR composite microspheres could be attributed to the following structural benefits: (1) The porous structure accommodated large volumetric deformations during the extended cycling, allowing smooth Na-ion diffusion due to the enhanced electrode wetting or electrolyte penetration; (2) The PDA-derived conductive C and NGC coated layer surrounding the active material facilitated the rapid charge transfer during the redox processes by enhancing the overall electrical conductivity. Similarly, the relatively better cycling performance of P-CoSe_2_@NGC NR than the bare CoSe_2_ hollow microspheres was attributed to the porous structure and thin NGC-coated layer surrounding the nanorods. However, P-CoSe_2_@NGC NR could not withstand the extreme stress caused by the large volume change of the active material during the long-term cycling, and eventually showed a steady decrease in capacity. In contrast, the bare CoSe_2_ hollow microspheres with a large crystallite size and no carbon layer did not accommodate the large volume change during the repeated Na-ion insertion process that consequently resulted in structural destruction and drastic capacity fading. Furthermore, the cycling performance using high-loading electrodes (1.5 mg cm^−2^) was also evaluated for P-CoSe_2_@PDA-C NR microspheres at a current density of 0.5 A g^−1^, as shown in Fig. S14. As observed, even with high loading electrodes, the sample display satisfactory cycling results. For instance, at 1.5 mg cm^−2^ loading, the discharge capacities were *ca.* 306 mAh g^−1^, respectively, at the end of 100th cycle, suggesting its practical applicability.

Further, to analyze the effect of PDA-derived carbon content and coating on the performance of P-CoSe_2_@PDA-C NRs, we performed the cycling stability tests (0.5 A g^−1^) for PDA amounts of 5 mg and 50 mg during the coating process. Prior to this, the coated samples with different PDA amounts (5, 10, and 50 mg) were analyzed. The HR-TEM images in Fig. S15a, c clearly indicates that with increase in PDA amount, the coating thickness increases concomitantly. For instance, with 5 mg PDA amount, the carbon layer thickness was 1.6 nm which increases to 8.1 nm when PDA amount was increased to 50 mg. The cycling performance results for different coating thickness samples are shown in Fig. S15d. The P-CoSe_2_@PDA-C NRs treated with a low PDA amount (5 mg) initially had the highest discharge capacity up to 50 cycles, which then continuously decreased and became unstable at high cycle numbers. This observation was attributed to the thin carbon coating layer that did not withstand the severe volume variation during the prolonged cycling. Similarly, the P-CoSe_2_@PDA-C NRs treated with a high PDA amount (50 mg) demonstrated stable cycling performance. However, the discharge capacity values were the lowest due to the high carbon content and coating that increased the effective diffusion lengths. These results firmly suggest that an optimized amount of PDA for carbon coating is crucial parameter for obtaining ultra-long-cycle stability performance and high reversible capacity.

The rate performance at various current densities ranging from 0.1 to 5.0 A g^−1^ was evaluated to examine the rate-capability tests of the prepared composite microspheres (Fig. 5e). The discharge capacities of P-CoSe_2_@PDA-C NR microspheres at current densities of 0.1, 0.2, 0.5, 0.7, 1.0, 1.5, 2.0, 3.0, 4.0, and 5.0 A g^−1^ were 380, 354, 303, 284, 261, 234, 212, 183, 156 and 133 mAh g^−1^, respectively, at the end of 10th cycle. A discharge capacity of 367 mAh g^−1^ (approximately 97% of the initial capacity) was recovered when the current density was reversed to 0.1 A g^−1^, suggesting excellent capacity retention and rate capability of P-CoSe_2_@PDA-C NR. In contrast, the discharge capacities of the P-CoSe_2_@NGC NR and bare CoSe_2_ hollow microspheres were 404/463, 367/358, 306/247, 277/169, 248/104, 215/52, 185/24, 141/9, 103/3, and 75/2 mAh g^−1^, respectively, at the identical current densities. The P-CoSe_2_@PDA-C NR microspheres had relatively low capacities due to the high carbon content for current densities below 0.5 A g^−1^ compared to other composites. However, the high-rate performance of the P-CoSe_2_@PDA-C NR validated its structural superiority as an advanced anode for NIBs compared with the P-CoSe_2_@NGC NR and bare CoSe_2_ hollow microspheres. The numerous porous channels in the nanostructure allow efficient electrode wetting, which promoted the fast redox processes during the electrochemical testing. Additionally, the synergetic effects of the NGC and PDA-derived C-coated conductive carbon enhanced the overall electrical conductivity of the electrode, which facilitated the rapid transfer of charge species by improving the electrical contact between the active sites of the CoSe_2_ nanorods constituting the P-CoSe_2_@PDA-C NR microspheres and the electrode. Table S3 compares the obtained electrochemical performance of the P-CoSe_2_@PDA-C NR microspheres in the present study with other reported cobalt-selenide nanostructures with various morphologies under the carbonate-based electrolyte system. From these observations, the P-CoSe_2_@PDA-C NR microspheres exhibited superior ultra-long cycling performance, suggesting long-lived advanced anodes for efficient Na-ion storage.

Furthermore, the capacity contribution of the carbon in the total capacity of P-CoSe_2_@NGC NR microspheres was quantified. The composite powders were soaked in highly concentrated (6.0 M) hydrochloric acid for 3 days to etch the Co and Se species, followed by repeated washing with distilled water and subsequent overnight drying at 100 °C in air. The physical and electrochemical results of the pure carbon microspheres without CoSe_2_ obtained after etching are summarized in Fig. S16. The FE-SEM micrographs (Fig. S16a) showed that the sample morphology was intact even after the etching process. Moreover, the CV curves obtained at 0.1 mV s^−1^ (Fig. S16b) confirmed the redox processes for carbon only, with a single sharp cathodic peak at 0.001 V corresponding to the Na intercalation in the carbon [50, 51]. The initial charge/discharge (Fig. S16c) and cycling performance (Fig. S16d) obtained at 0.5 A g^−1^ further supported these results. The cycling performance suggested that the pure carbon had a low discharge capacity of approximately 52 mAh g^−1^ at the 100th cycle, approximately 15% of the P-CoSe_2_@NGC NR microspheres at the identical current density and cycle number (Fig. 5c). Similarly, the pure carbon had a discharge capacity of 22 mAh g^−1^ after the 100th cycle at a current density of 2.0 A g^−1^ (Fig. S16e), indicating a capacity contribution of *ca.*11%. Therefore, the above results confirm that carbon only acts as a conductive scaffold for the rapid charge transfer during the electrochemical process.

**Fig. 6:**
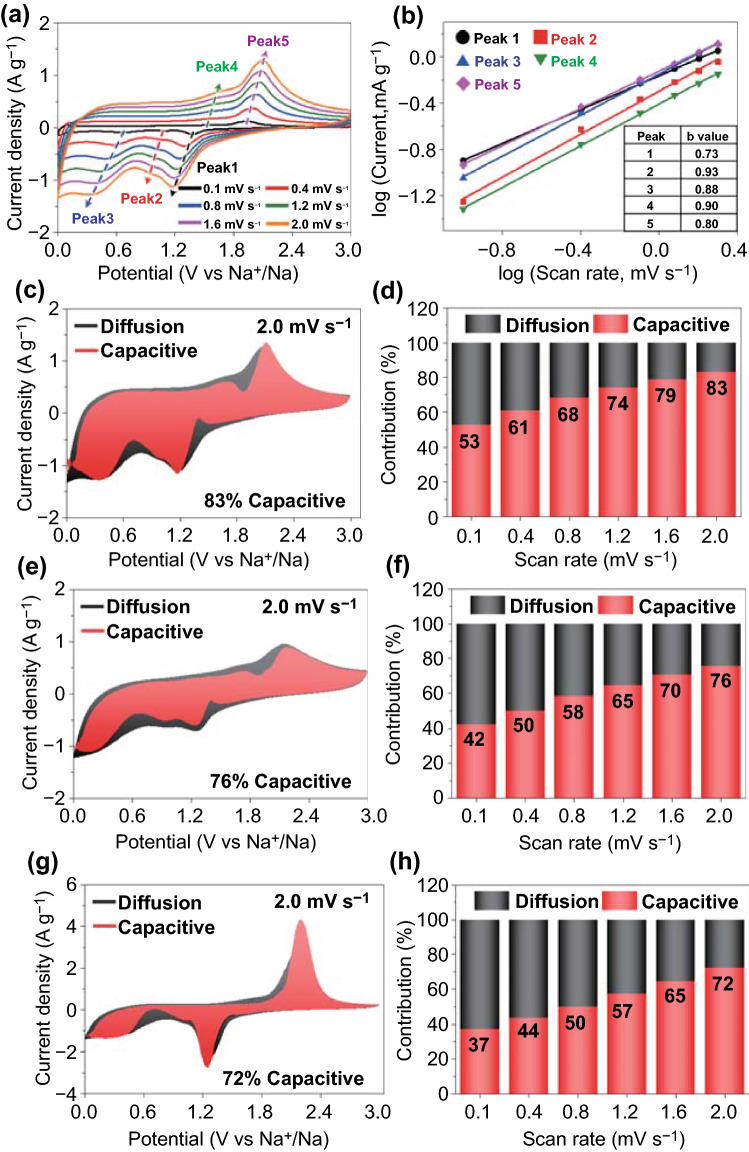
Electrochemical reaction dynamics analysis of **a**–**d** the P-CoSe_2_@PDA-C NR, **e**, **f** P-CoSe_2_ @NGC NR, and **g**, **h** bare CoSe_2_ hollow microspheres: **a** CV curves obtained at various scan rates, **b** current response (*i*) versus scan rate (*n*) at each redox peak, **c**, **e**, **g** CV curves with the capacitive fraction shown by the red region at a scan rate of 2.0 mV s^−1^, and **d**, **f**, **h** bar charts showing the percentage of the capacitive contribution at different scan rates

To better understand the electrochemical kinetics inside the cell, the CV curves are plotted for P-CoSe_2_@PDA-C NR microspheres in the voltage window of 0.001–3.0 V at different scan rates, as shown in Fig. 6a. Subsequently, the graphs were plotted between the peak current (*i*) during reduction/oxidation and the scan rates (*v*) according to the following power law relation to differentiate between the capacitive- and diffusion-controlled processes in the CV curves [62, 63]:

5 $$i=a{v}^{b}$$

6 $$\mathrm{log}\left(i\right)=b\mathrm{log}\left(v\right)+\mathrm{log}\left(a\right)$$

here, variables *a* and *b* determined whether the process was diffusion- or capacitive-controlled. The redox process was capacitive when *b* approached 1.0 and diffusion-controlled when *b* approached 0.5 [62, 63]. The *b* values were determined using the slope of the log(*i*) vs. log(*v*) plots for different cathodic and anodic peaks. The calculated *b* values at different redox peaks for the samples are shown in Figs. 6b and S14. The *b*-values for the five different redox peaks in the P-CoSe_2_@PDA-C NR microspheres were 0.73, 0.93, 0.88, 0.90, and 0.80, indicating a capacitive-dominant process. In contrast, the reaction dynamics for the P-CoSe_2_@NGC NR microspheres shown in Fig. S17a, b demonstrate lower *b* values, suggesting a marginally lower capacitive-controlled redox process. Similarly, in Fig. S17c, d, the bare CoSe_2_ hollow microspheres exhibit the lowest *b* values among the samples, indicating that a capacitive process continued to govern the redox processes only to a lower extent. Generally, the capacitive effect of the electrode material is closely related to the reaction kinetics, implying that the transport kinetics is enhanced when the percentage of the capacitive-controlled process is higher. Therefore, the total stored charge in the electrode material was separated into capacitive- and diffusion-controlled processes for the quantitative analysis of the capacity contribution to the current response using the following equation [64, 65]:

7 $$i={k}_{1}v+{k}_{2}{v}^{1/2}$$

where *k*_1_*v* and *k*_2_*v*^1/2^ are the capacitive and diffusion contributions, respectively, while *k*_1_ and *k*_2_ are the constants obtained from the slope and intercept of the *i*(V)/*v*^1/2^ versus *v*^1/2^ plot, respectively [64, 65]. As shown in Fig. 6c, the capacitive contribution factor (*k*_1_*v*) for the P-CoSe_2_@PDA-C NR microspheres, highlighted by the red region, is 88% at a scan rate of 2.0 mV s^−1^. A relatively high capacitive contribution was observed for the P-CoSe_2_@PDA-C NR microspheres at all scan rates, excluding 2.0 mV s^−1^ (Fig. 6d). In contrast, the surface-controlled reaction contributions in P-CoSe_2_@NGC NRs (Fig. 6e, f) had a lower percentage of the capacitive process even at a high scan rate of 2.0 mV s^−1^ (76%). Similarly, the bare CoSe_2_ hollow microspheres in Fig. 6g, h demonstrates similar results with low capacitive processes at high and low scan rates. These results confirm the kinetically favored rapid Na-ion transport for P-CoSe_2_@PDA-C NR microspheres owing to the better electrolyte percolation through the porous structure, efficient mitigation of volume variation during cycling, and synergetic effects of two highly conductive carbon-coated layers that facilitate rapid charge transfer.

**Fig. 7:**
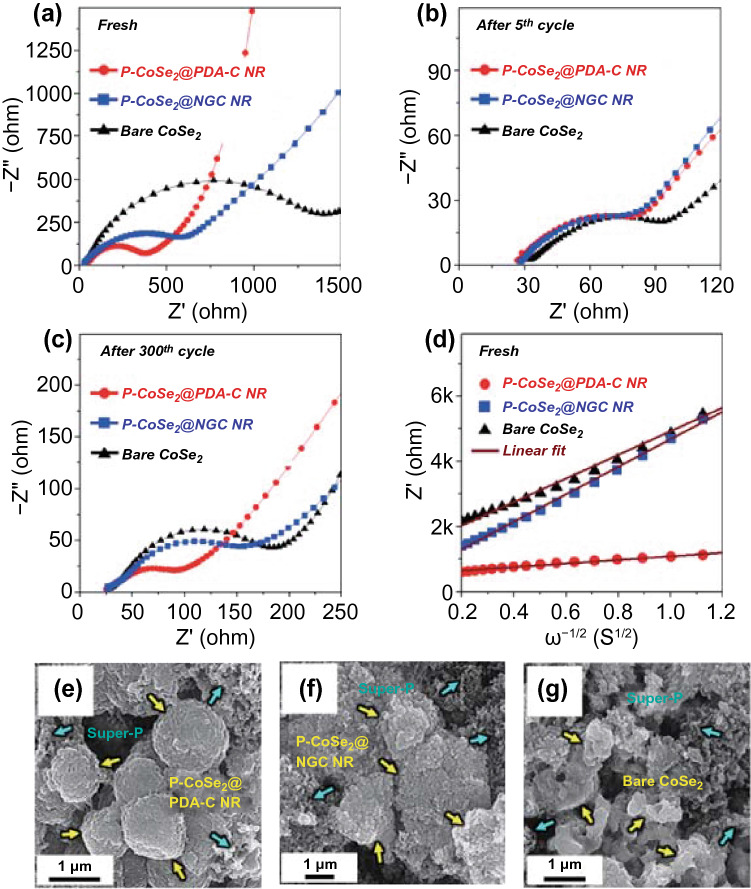
**a**–**d** Nyquist impedance plots and **e**–**g** FE-SEM images of the P-CoSe_2_@PDA-C NR, P-CoSe_2_ @NGC NR, bare CoSe_2_ hollow microspheres obtained after 300th cycles at 0.5 A g^−1^: **a** before cycling, **b** after 5th cycle, **c** after 300th cycle, **d** relationships between the real part of the impedance (Z_re_) and *ω*^−1/2^ of the samples before cycling, **e** the P-CoSe_2_@PDA-C NR, **f** P-CoSe_2_ @NGC NR, and **g** bare CoSe_2_ hollow microspheres

EIS measurements were carried out using the deconvolution Randle-type equivalent-circuit model of the cell shown in Fig. S18, and the fitted values of the three prepared structures are summarized in Table S4. The Nyquist plots for the fresh cells (Fig. 7a) had marginally different electrolyte resistance (*R*_e_) values (30–35 Ω), indicating slightly different electrode–electrolyte interface reactions. Furthermore, the charge transfer resistance (*R*_ct_) values of the P-CoSe_2_@PDA-C NR, P-CoSe_2_@NGC NR, and bare CoSe_2_ hollow microspheres were 362, 755, and 1463 Ω, respectively (Fig. 7a). The large crystallite size of the bare CoSe_2_ hollow microspheres results in an ultra-high *R*_ct_ value of the cell. However, a substantial decrease in the *R*_ct_ (P-CoSe_2_@PDA-C NR: 81 Ω, P-CoSe_2_@NGC NR: 88 Ω, and bare CoSe_2_ hollow microspheres: 83 Ω) was observed for all samples after five cycles due to the formation of ultrafine CoSe_2_ nanocrystals in the structure after the first cycle (Fig. 7b) [66–68]. After the 300th cycle (Fig. 7c), the P-CoSe_2_@PDA-C NR had the lowest *R*_ct_ value of 76 Ω than the P-CoSe_2_@NGC NR (152 Ω) and bare CoSe_2_ hollow microspheres (168 Ω), suggesting fast redox kinetics and superior electrode integrity of the P-CoSe_2_@PDA-C NR microspheres. To further analyze the Nyquist plots, Z_re_ as a function of *ω*^−1/2^ (*ω* = 2πf is the angular frequency) is plotted for the low-frequency region, as shown in Fig. 7d. The less steep slope at low frequencies for the P-CoSe_2_@PDA-C NR microspheres indicated higher Na-ion diffusivity in the structure than the P-CoSe_2_@NGC NR and bare CoSe_2_ hollow microspheres, thus confirming the electrochemical results. Furthermore, the Na-ion diffusion coefficient (${D}_{{Na}^{+}}$) values were calculated from Fig. 7d using the following equation [35, 69, 70]:

8 $${D}_{{Na}^{+}}=\frac{0.5{R}^{2}{T}^{2}}{{A}^{2}{F}^{4}{C}^{2}{\sigma }_{W}^{2}}$$

where ${D}_{{Na}^{+}}$ is the Na-ion diffusion coefficient, *R* is the gas constant, *T* is the temperature, *A* is the electrode area, *C* is the Na-ion concentration, *F* is the Faraday constant, and ${\sigma }_{W}$ is the Warburg impedance factor. The ${D}_{{Na}^{+}}$ values for the P-CoSe_2_@PDA-C NR, P-CoSe_2_@NGC NR, and bare CoSe_2_ hollow microspheres were 4.61 × 10^−14^, 0.79 × 10^−15^, and 1.07 × 10^−15^ cm^2^ s^−1^, respectively. Thus, the ${D}_{{Na}^{+}}$ value of the P-CoSe_2_@PDA-C NR microspheres was one order of magnitude higher than that of the other microsphere, indicating much faster Na-ion diffusion and thereby superior redox reaction kinetics during the charge–discharge process. The higher diffusion values for P-CoSe_2_@PDA-C NR microspheres confirmed the structural robustness of the prepared sample that could withstand the ultra-long cycling life and maintain the mechanical integrity of the electrode. Besides, the marginally better ${D}_{{Na}^{+}}$ for bare CoSe_2_ compared to the P-CoSe_2_@NGC NR sample was due to its high crystalline nature. However, the ${D}_{{Na}^{+}}$ coefficient was also calculated using the EIS data at the end of 300th cycle. The diffusion coefficient values for the P-CoSe_2_@PDA-C NR, P-CoSe_2_@NGC NR, and bare CoSe_2_ hollow microspheres comes out to be 4.72 × 10^−14^, 2.46 × 10^−14^, and 9.78 × 10^−15^ cm^2^ s^−1^ (Fig. S19). Therefore, the ${D}_{{Na}^{+}}$ values are one order higher for P-CoSe_2_@NGC NR compared to the bare CoSe_2_ hollow microspheres. This result again confirms that the inferior cycling performance of bare CoSe_2_ is mainly due to poor Na-ion diffusion. The morphologies of all the cycled electrodes obtained after the 300th cycle (0.5 A g^−1^) are shown in Fig. 7e–g to confirm the structural integrity of the P-CoSe_2_@PDA-C NR. The FE-SEM images of the P-CoSe_2_@PDA-C NR (Fig. 7e) after cycling indicated that the microspheres maintained a spherical morphology, implying the high structural integrity of the prepared microspheres. Furthermore, the formation of SEI film on the surface was confirmed through XPS analysis of P-CoSe_2_@PDA-C NR microspheres after 300th cycling (Fig. S20 and the corresponding discussion). The spherical morphology of the P-CoSe_2_@NGC NR microspheres (Fig. 7f) began to collapse owing to their inability to accommodate the volume change fully during the prolonged cycling. Similarly, the bare CoSe_2_ hollow microspheres (Fig. 7g) revealed the complete degradation of the structure into a highly aggregated powder-type morphology due to their non-porous structure. These results suggest that the structural advantages in P-CoSe_2_@PDA-C NR microspheres driven by a highly porous structure and the synergetic effects of NGC and PDA-derived C-coated layer provide exceptional mechanical integrity to the electrode that subsequently resulted in overwhelming electrochemical performance. Overall, we believe that the synthesis of porous microspheres with two kinds of carbon-coated layers as a highly stable and conductive scaffold resulted in high structural stability that can tolerate the severe volume variation during the repeated cycling and provide conductive pathways for faster Na-ion transfer, leading to superior electrochemical performance.

## Conclusion

In summary, we developed highly conductive and porous 3D microspheres uniformly deposited by the MOF-templated NGC and PDA-derived C-coated 1D CoSe_2_ nanorods (P-CoSe_2_@PDA-C NR microspheres) as building blocks using scalable spray-drying process, followed by the post-heat-treatment and facile coating techniques. The porous structure derived by the PS nanobead suspension provides enormous space to channel the extreme volume variations and efficient electrolyte filtration that guarantees fast diffusion of charge species. Moreover, the synergetic effects of dual carbon coating arrangement, NGC and PDA-derived C-coating, provide numerous conductive primary and secondary transport pathways that allow the rapid transfer of charge species to support the fast redox processes. Benefitted from the structural merits, the P-CoSe_2_@PDA-C NR microspheres exhibit exceptional ultra-long cycling stability and reasonable rate capability. Therefore, we believe that the structural advancements presented in this study can benefit the research community in visualizing highly conductive and porous nanostructures for long-lived electrodes in various energy storage applications.

## Supplementary Information

Below is the link to the electronic supplementary material.

Supplementary file1 (PDF 2103 KB)
