# Supplementary material for: Porous Microspheres Comprising CoSe2 Nanorods Coated with N-Doped Graphitic C and Polydopamine-Derived C as Anodes for Long-Lived Na-Ion Batteries
Source: Nanomicro Lett. 2022 Apr 28;14:113. doi: 10.1007/s40820-022-00855-z (PMC9050979; doi:10.1007/s40820-022-00855-z)
Supplement: Supplementary file 1 — Supplementary file1 (PDF 2103 KB) [file 40820_2022_855_MOESM1_ESM.pdf]

Supporting Information for

Porous Microspheres Comprising $CoSe_2$ Nanorods Coated with N-Doped Graphitic C and Polydopamine-Derived C as Anodes for Long-Lived Na-Ion Batteries

Jae Seob Lee[1], Rakesh Saroha[1], and Jung Sang Cho[1,*]

[1]Department of Engineering Chemistry, Chungbuk National University, Chungbuk 361-763, Republic of Korea

*Corresponding author. E-mail: jscho@cbnu.ac.kr (Jung Sang Cho)

Supplementary Figures and Tables

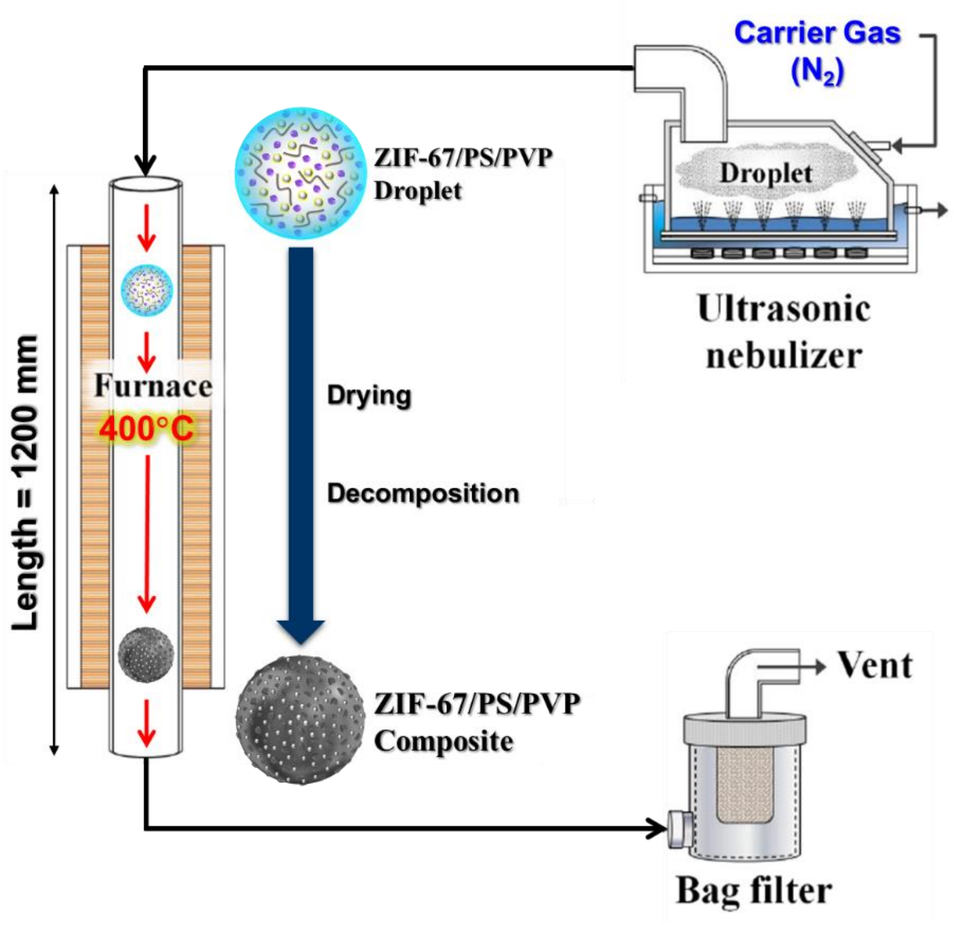

Scheme S1 Schematic diagram of the spray pyrolysis system for the preparation of ZIF-67/PS/PVP composited precursor microspheres

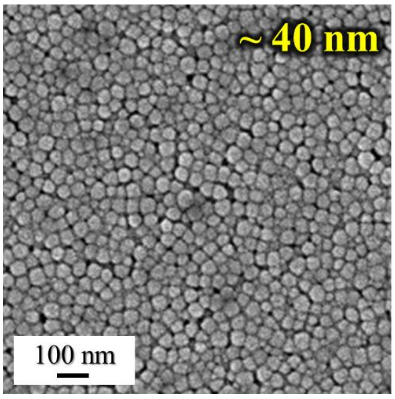

Fig. S1 FE-SEM image of PS nanobeads used to prepare spray solution

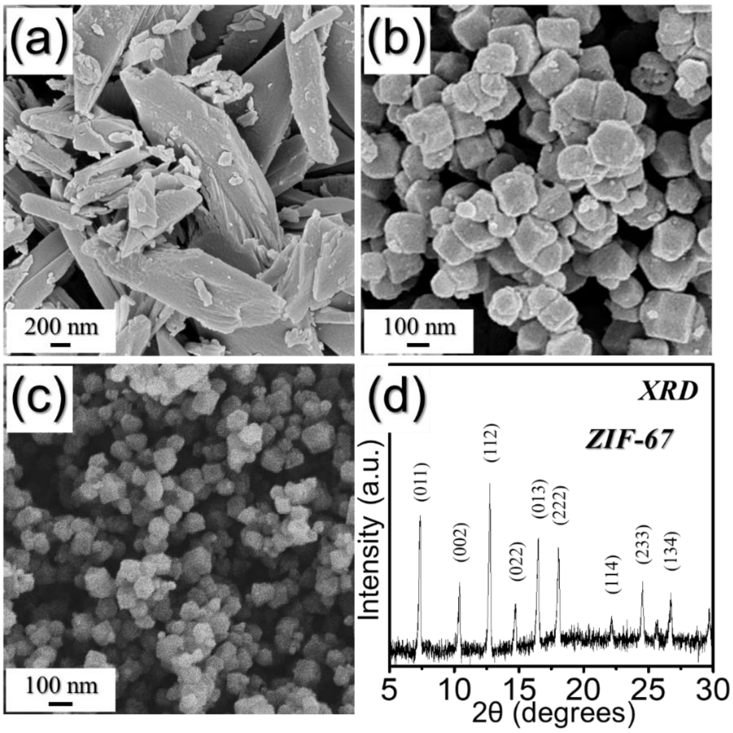

Fig. S2 Physical characteristics of the prepared ZIF-67 polyhedrons obtained from the solution **a** without TEA, with **b** 3 mL TEA, **c** 6 mL TEA, and **d** XRD pattern of ZIF-67 polyhedrons with 6 mL TEA

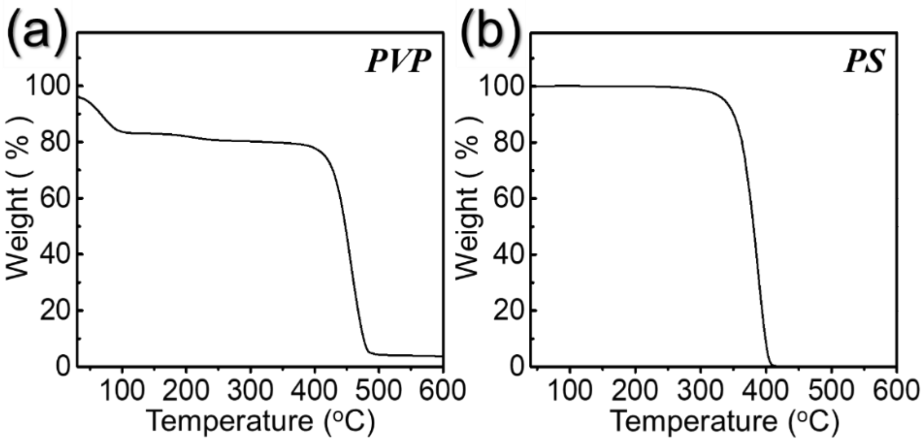

Fig. S3 TG curves of **a** polyvinylpyrrolidone (PVP), and **b** polystyrene (PS)

The pores formed by the decomposition of PS nanobeads has both roles on the formation of $CoSe_2$ nanorods during the selenization process. Firstly, the pores facilitate the penetration of H_2Se gas into the structure, promoting uniform selenization, both inside and on the external surface. Secondly, the pores also guarantee the enough space between nanorods that prohibits the agglomeration of $CoSe_2$ nanorods and allow them to grow. To confirm this reasoning, the ZIF-67/PVP microspheres without PS beads is prepared and analyzed the morphological changes after selenization at different temperatures, as shown Fig. S4. As observed, the $CoSe_2$ rods start to form at external surface. As the reaction proceeds, they sintered into non-rod like particles due to the absence of pores or enough space between nanorods to prevent agglomeration.

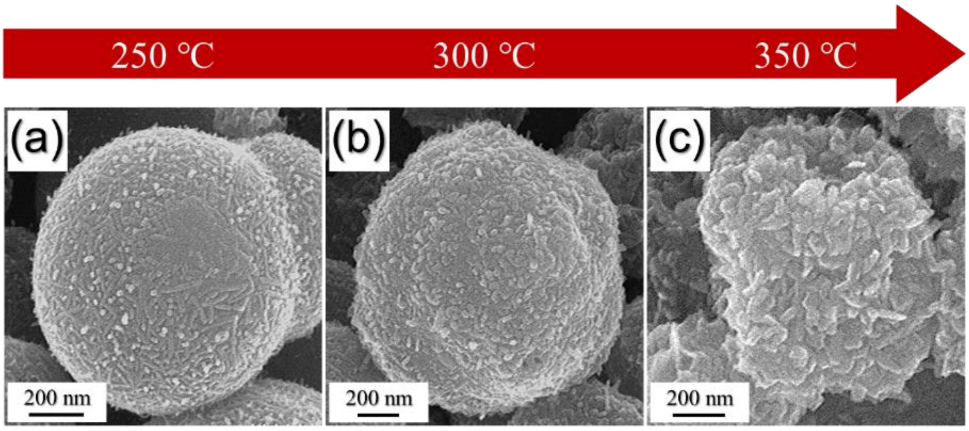

Fig. S4 FE-SEM images of ZIF-67/PVP composite microspheres without PS nanobeads after selenization at different temperatures: **a** 250 °C, **b** 300 °C, and **c** 350 °C

The presence of PS nanobeads in the composite microspheres and their subsequent decomposition guarantees in highly porous 3D microspheres with efficient conversion of central Co atom in ZIF-67 to 1D $CoSe_2$ NR during the selenization process due to the effective penetration of H_2Se gas and guaranteed the enough space between nanorods to prevent agglomeration. In this regard, it is highly indispensable to elucidate the effect of PS nanobeads on the formation of porous microspheres covered with $CoSe_2$ nanorods. The crystal structure and morphological features of the as-sprayed ZIF-67/PVP (i.e., without PS nanobeads suspension) composite microspheres prepared under identical conditions are shown in Fig. S5 along with the detailed synthesis mechanism (Fig. S5a). The FE-SEM image of the ZIF-67/PVP composite microsphere (Fig. S5b) also displayed spherical morphology with a mean diameter of about 1.0 µm. The lower dimension of the microsphere seems obvious due to the absence of PS nanobeads. The as-sprayed composite microspheres were further subjected to selenization process, and the morphology of resulted microspheres is presented in Fig. S5c. The FE-SEM image clearly indicates highly aggregated non-rod like particles due to insufficient space between nanorods, which covered the microsphere (mean diameter ~0.8 µm) surface suggesting incomplete directional recrystallization process in the non-porous structure. Besides, the XRD pattern in Fig. S5d indicates sharp diffraction peaks that could be assigned to o-$CoSe_2$ crystal structure.

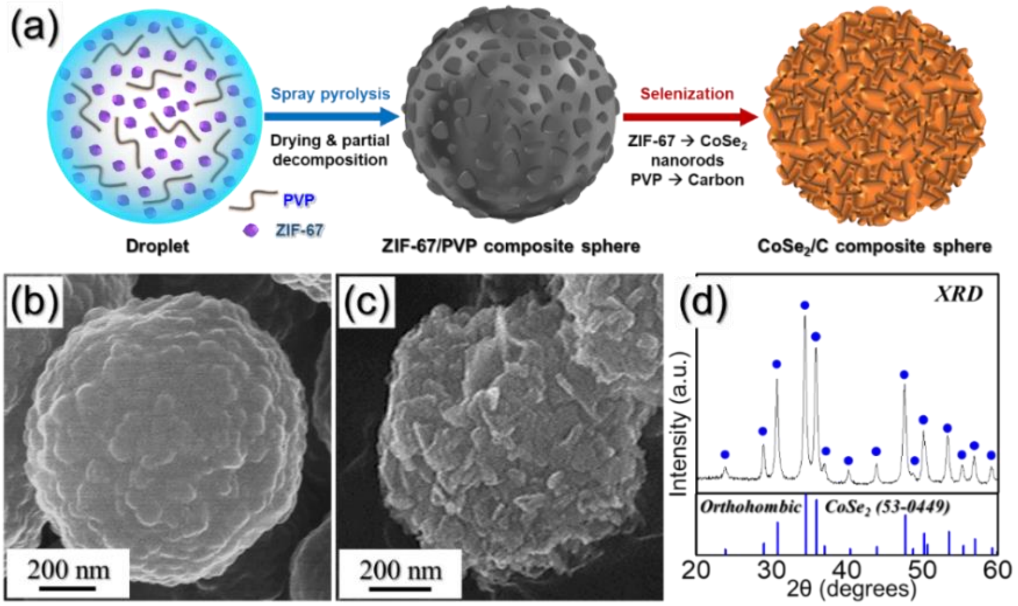

Fig. S5 **a** Schematic representation of formation mechanism of the $CoSe_2$/C composite microspheres, **b** FE-SEM images of the ZIF-67/PVP composite microspheres prepared without PS nanobeads, **c** FE-SEM images of the $CoSe_2$/C composite microspheres obtained after selenization at 350 °C, and **d** XRD pattern of the $CoSe_2$/C composite microspheres

Table S1 Inductively coupled plasma-optical emission spectrometer (ICP-OES) results for P-$CoSe_2$@NGC NR microspheres

Sample	Cobalt (wt%)	Selenium (wt%)
**P-$CoSe_2$@NGC NR**	20.4	57.6

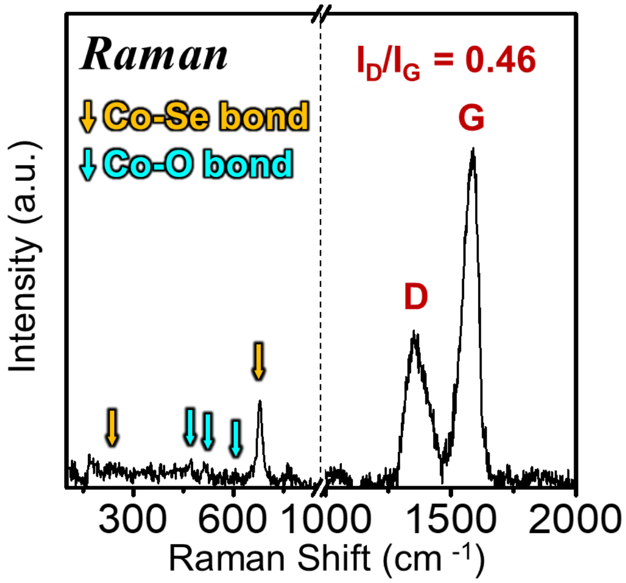

Fig. S6 Raman spectrum of the P-$CoSe_2$@NGC NR microspheres

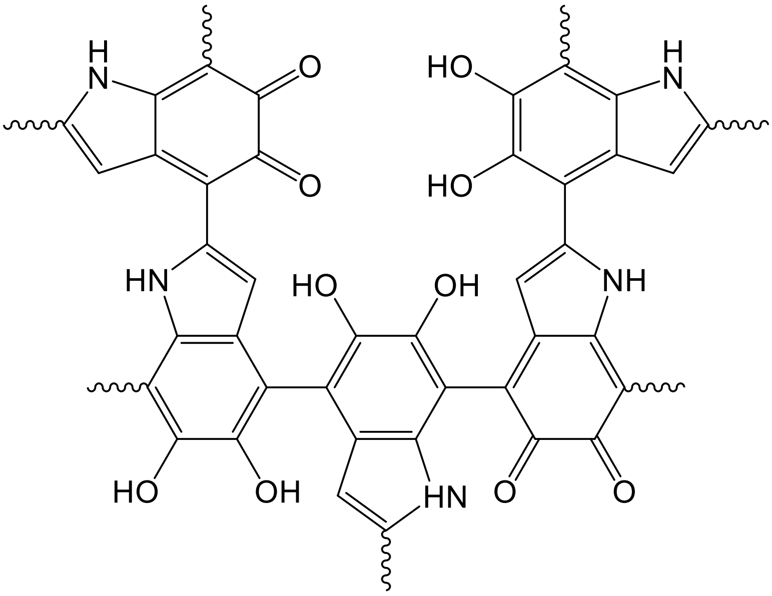

Fig. S7 The molecular structure of polydopamine (PDA)

Table S2 Elemental analysis (EA) of the P-$CoSe_2$@NGC NR microspheres and P-$CoSe_2$@PDA-C NR microspheres

Sample	Carbon (wt%)	Nitrogen (wt%)
**P-$CoSe_2$@NGC NR**	16	2
**P-$CoSe_2$@PDA-C NR**	33	4

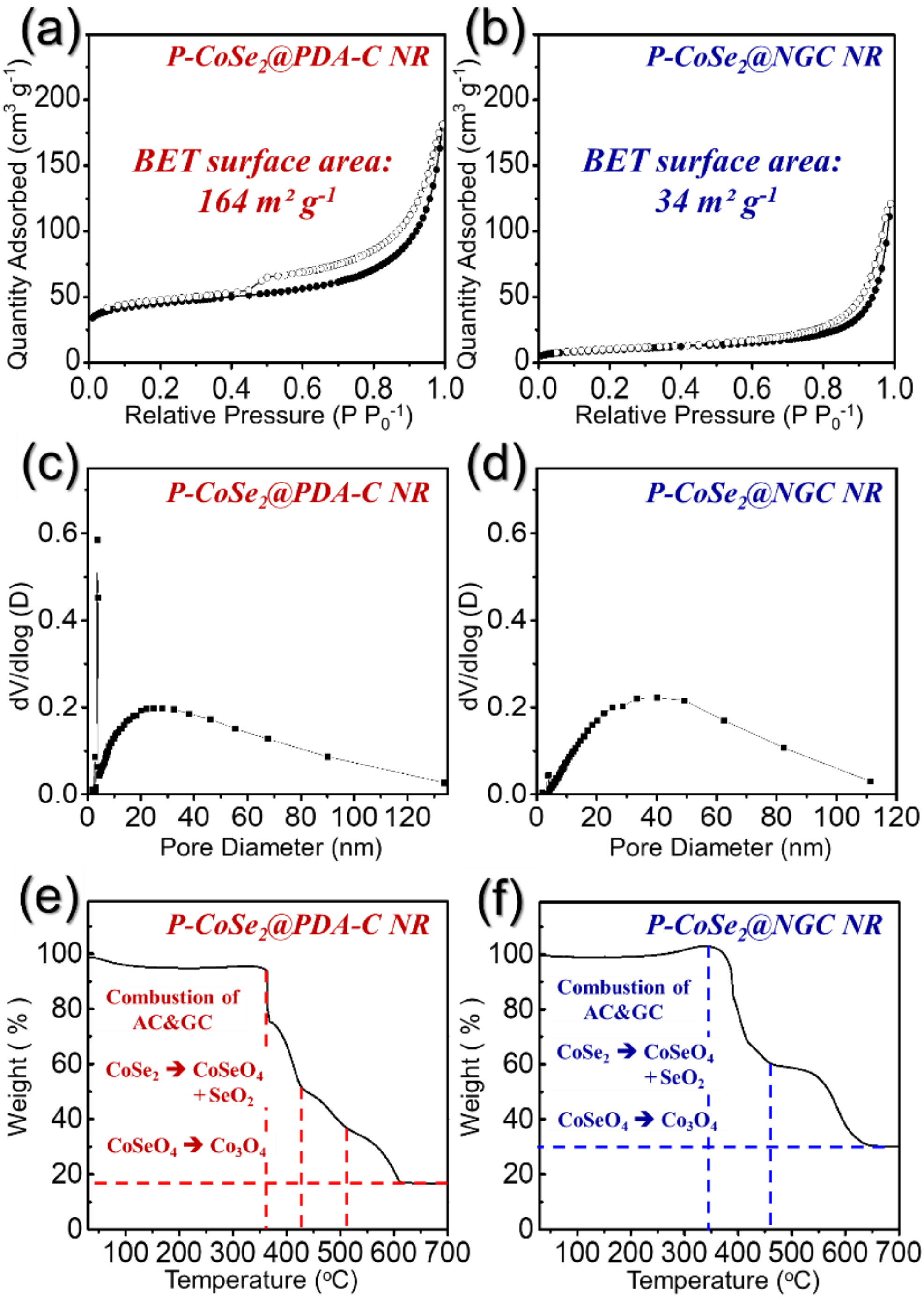

Fig. S8 a, b N_2 adsorption-desorption isotherms, **c,d** BJH desorption pore-size distributions and **e, f** TG curves of samples: **a, c, e** P-$CoSe_2$@PDA-C NR and **b, d, f** P-$CoSe_2$@NGC NR microspheres

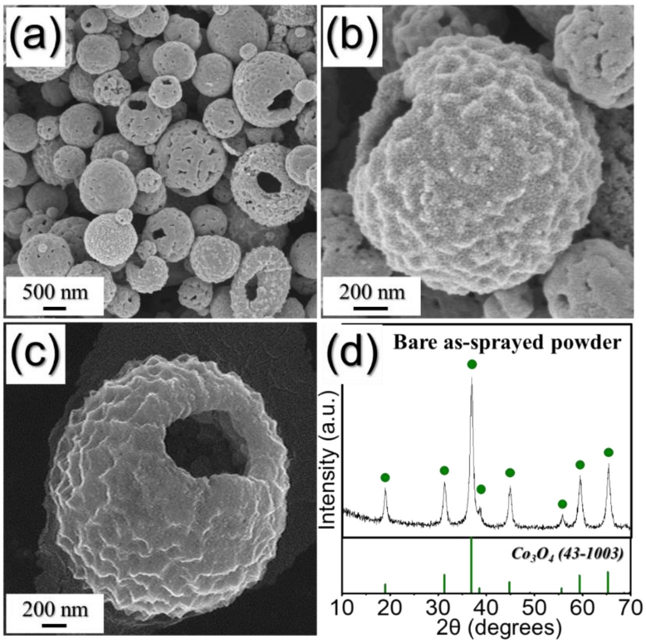

Fig. S9 a-c FE-SEM images, and **d** XRD pattern of the bare Co_3O_4 hollow microspheres obtained after spray pyrolysis in an air atmosphere at 400 °C

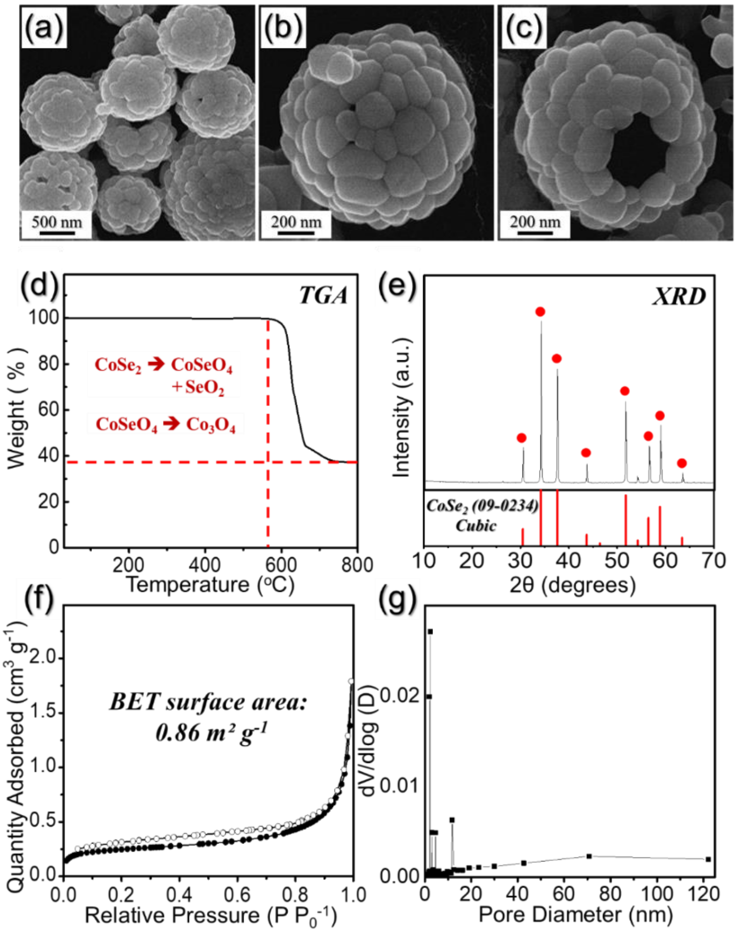

Fig. S10 a-c FE-SEM images, **d** TG curve, **e** XRD pattern, **f** N_2 adsorption-desorption isotherms, and **g** BJH desorption pore-size distributions of the bare $CoSe_2$ hollow microspheres

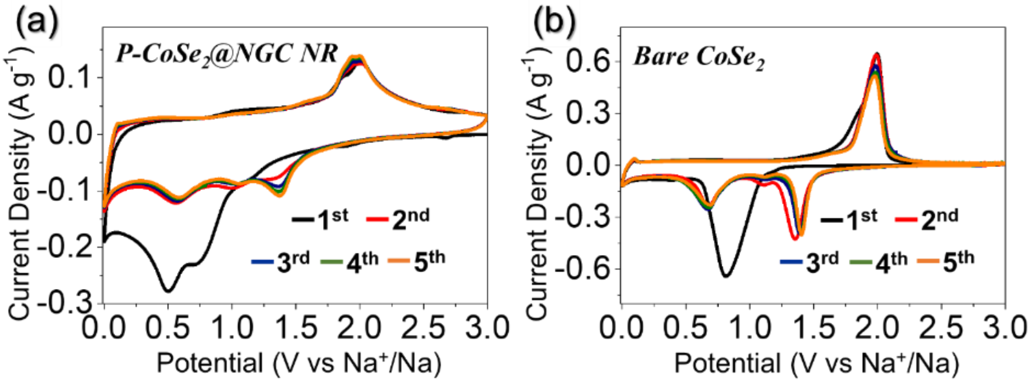

Fig. S11 Cyclic voltammogram (CV) curves of **a** the P-$CoSe_2$@NGC NR microspheres and **b** bare $CoSe_2$ hollow microspheres

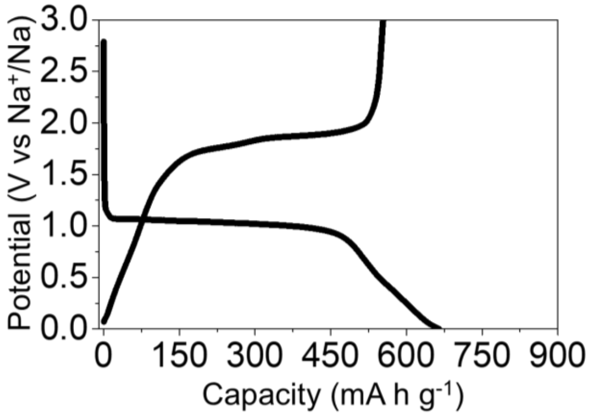

Fig. S12 Initial charge/discharge curves of the bare $CoSe_2$ hollow microspheres at a current density of 0.1 A g^{-1}

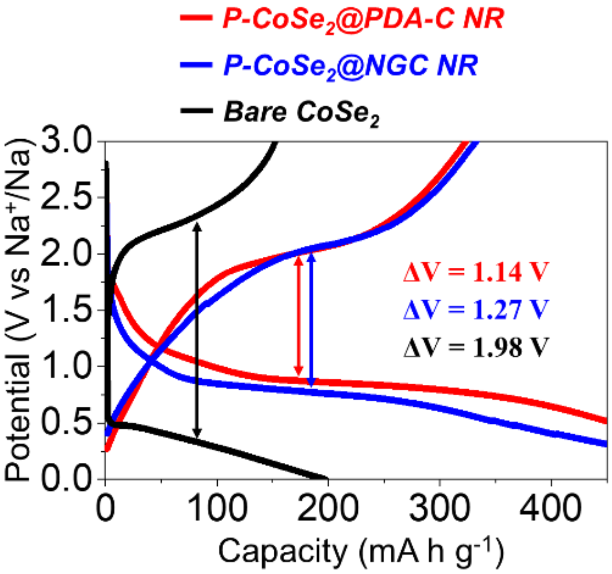

Fig. S13 Initial discharge/charge curves at a current density of 0.5 A g^{-1}

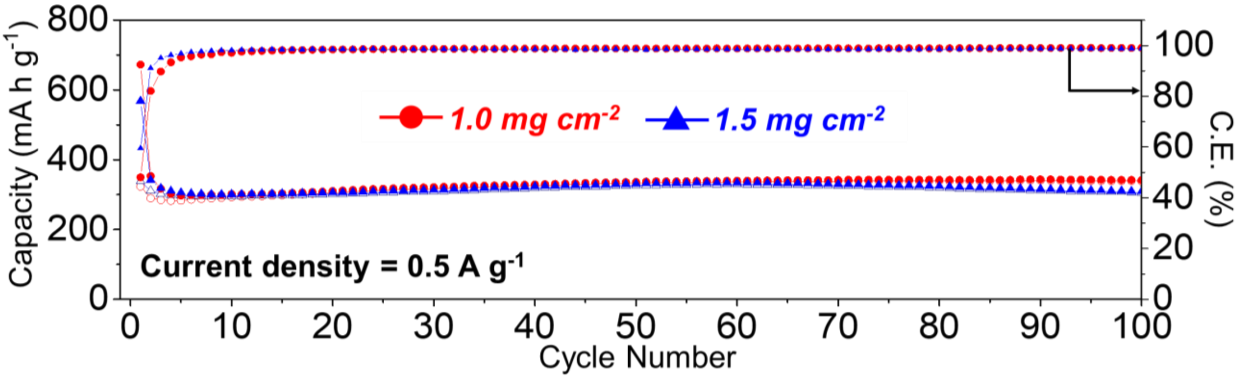

Fig. S14 Cycling performance of the P-$CoSe_2$@PDA-C NR microspheres with different mass loading of active material at a current density of 0.5 A g^{-1}

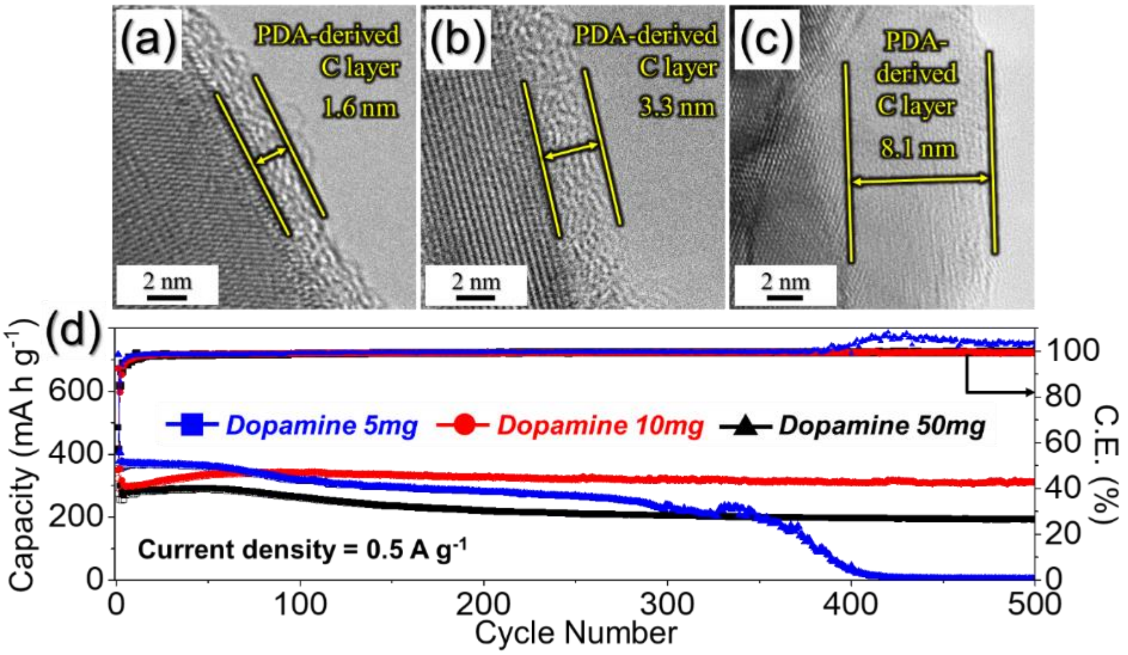

Fig. S15 a-c HR-TEM images and **d** cycling performance of P-$CoSe_2$@PDA-C NR microspheres synthesized with different amounts of dopamine hydrochloride at a current density of 0.5 A g^{-1}: **a** 5 mg, **b** 10mg, **c** 50mg

Table S3 Electrochemical performance comparison of P-$CoSe_2$@PDA-C NR microspheres prepared in this study with other cobalt selenides as anodes reported previously

Materials	Current Density [A g^{-1}]	Initial C_{dis}/C_{cha} [mA h g^{-1}] /ICE [%]	Cycle No./ Discharge Capacity [mA h g^{-1}]	Capacity Retention [%]	Average Discharge Potential [V]	Mass Loading [mg cm^{-2}]	Refs.
**P-$CoSe_2$@PDA-C NR**	**0.5**<br>**2.0**	**640/308/48**<br>**515/287/56**	**1,000/291**<br>**5,000/142**	**92**<br>**75***	**0.9**	**1.0**	**In this work**
$CoSe_2$@N-doped C@TiO_2 nanoparticle	0.2	645/488/76	200/374	78	-	0.88	[S1]
$CoSe_x$-rGO composite powder	0.3	656/459/70	50/420	80	-	1.2	[S2]
$CoSe_2$@N-doped porous graphitic carbon/CNT composite	0.2	601/454/76	100/424	-	-	1.2	[S3]
$CoSe_2$@N-doped carbon ramework/CNT composite	1.0	713/531/74	100/499	92	-	2.0	[S4]
$CoSe_2$@N-doped carbon nanorod/CNT porous composite microsphere	0.2	926/609/66	100/555	85	-	1.3	[S5]
Bone-like $CoSe_2$ nano-thorn coated on porous carbon cloth	0.1	1260/553 /44	180/361	74	-	0.5	[S6]
Cu-doped $CoSe_2$ microbox	1.0	-	500/365	94	-	-	[S7]
Necklace like-CNT/$CoSe_2$@ N-doped carbon composite particle	0.2	686/462/67	120/404	80	-	1.2	[S8]
$CoSe_2$@N-doped carbon matrix	1.0	-	500/282	-	-	-	[S9]

"* : Measured after stabilization (150 cycles)"

Note: It should be noted that all the references in the comparision table are based on **carbonate-based elctrolyte system**.

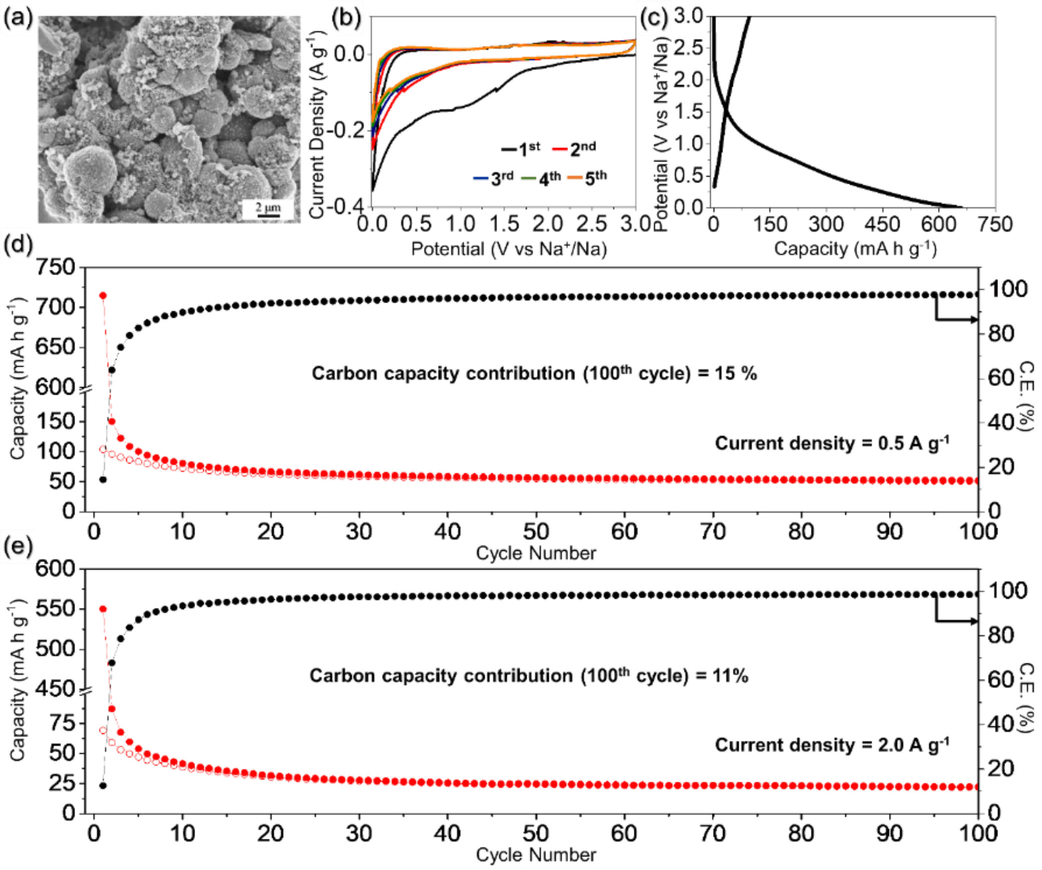

Fig. S16 Physical and electrochemical characterizations of PDA derived conductive C/NGC microspheres obtained from etched P-$CoSe_2$@PDA-C NR microspheres using hydrochloric acid: **a** FE-SEM image, **b** cyclic voltammetry curves at 0.1 mV s^{-1}, **c** initial discharge/charge profile, **d** cycling performance at 0.5 A g^{-1}, and **e** cycling performance at 2.0 A g^{-1}

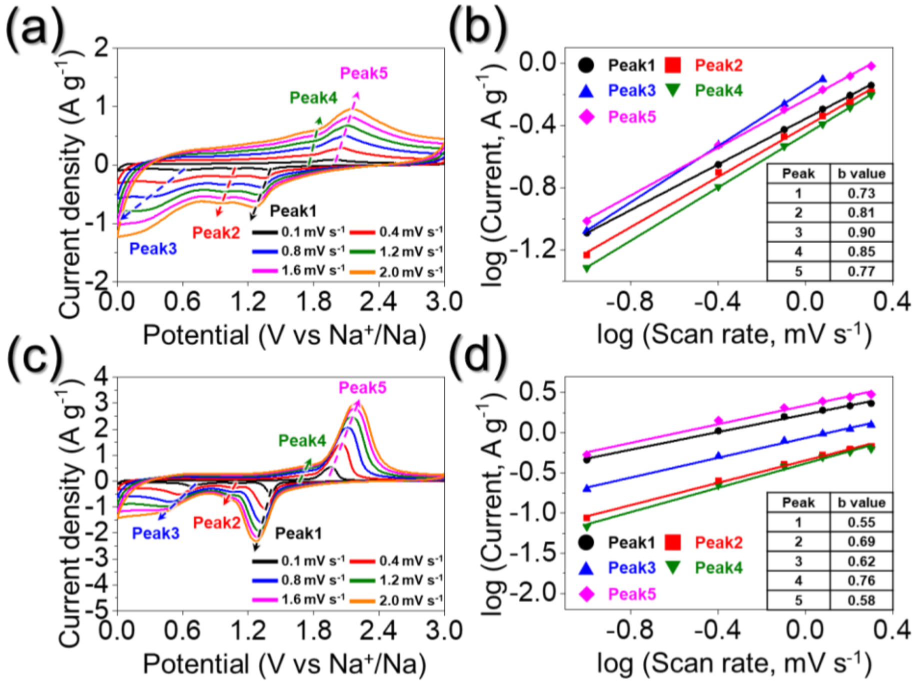

Fig. S17 Electrochemical reaction dynamics analysis of **a** and **b** the P-$CoSe_2$@NGC NR microspheres and **c** and **d** bare $CoSe_2$ hollow microspheres: **a and c** CV curves obtained at various scan rates, **b** and **d** current response (*i*) vs. scan rate (*n*) at each redox peak

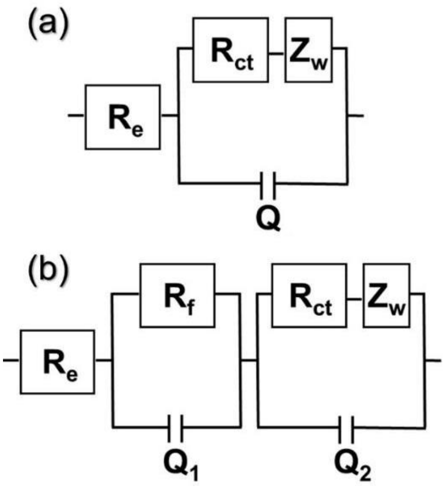

Fig. S18 Equivalent circuit model used for AC impedance fitting: **a** before cycling, and **b** after cycling, R_{ct} = charge-transfer resistance, R_e = electrolyte resistance, R_f = SEI layer resistance, Q_1 = dielectric relaxation capacitance, Q_2 = associated double layer capacitance

Table S4 EIS fitted parameters for each Na-ion cells utilizing three samples as anode materials before cycle, after 5th, and after 300th cycle at 0.5 A g^{-1}

Sample	Fresh cell			After 5th cycle					After 300th cycle				
	R_e (Ω)	R_{ct} (Ω)	Q	R_e (Ω)	R_f (Ω)	Q_1	R_{ct} (Ω)	Q_2	R_e (Ω)	R_f (Ω)	Q_1	R_{ct} (Ω)	Q_2
**P-$CoSe_2$@PDA-C NR**	30	362	0.71	27	11	0.73	81	0.66	25	29	0.62	76	0.69
**P-$CoSe_2$@NGC NR**	30	755	0.58	28	16	0.54	88	0.61	26	51	0.64	152	0.73
**Bare $CoSe_2$**	35	1463	0.66	33	18	0.61	83	0.63	28	73	0.68	168	0.79

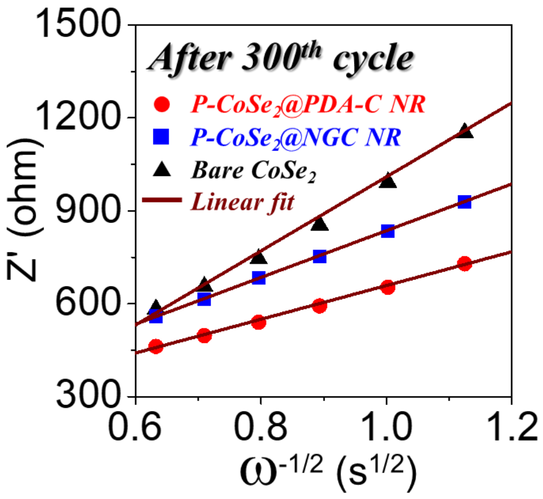

Fig. S19 Relationship between the real part of the impedance (Z_{re}) and $\omega^{-1/2}$ of the P-$CoSe_2$@PDA-C NR, P-$CoSe_2$@NGC NR, and bare $CoSe_2$ hollow microspheres obtained after 300th cycles at 0.5 A g^{-1}

The XPS analysis for the cycled electrode (P-$CoSe_2$@PDA-C NR microspheres after 300 cycles) is performed to confirm the presence of SEI layer, as shown below. As observed, the XPS survey spectrum shown in Fig. S20a not only exhibits Co 2p, Se 3d, C 1s, and N 1s signals but also display photoelectron signal attributed to Na 1s at a binding energy of 1071.6 eV [S10, S11]. The presence of high intense Na 1s peak strongly indicates the existence of Na_2CO_3 that formed due to electrolyte decomposition and subsequent formation of solid electrolyte interface, as reported previously (Fig. S20b) [S10, S11]. Likewise, the deconvoluted C 1s spectrum (Fig. S20c) also indicates the presence of various bonds that corresponds to different species usually present in SEI [S12-S14]. Based on the above observations, the authors believed that the SEI was formed in the cycled electrodes.

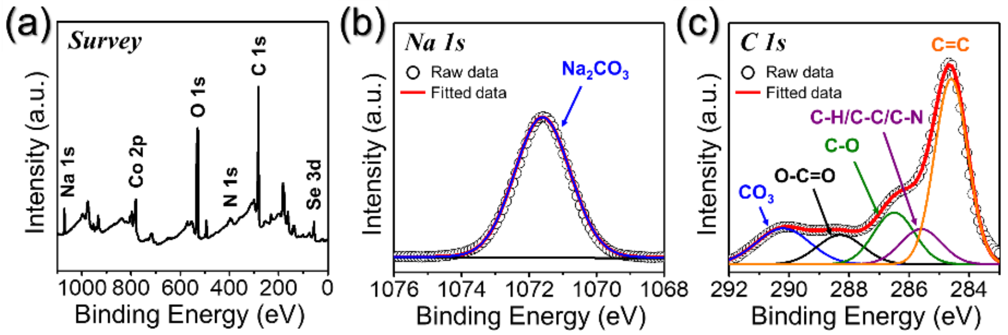

Fig. S20 XPS survey spectrum, and core-level XPS spectra: **a** XPS survey spectrum, **b** Na 1s, and **c** C 1s of the P-CoSe2@PDA-C NR microspheres obtained after 300th cycles at 0.5 A g^{-1}

Supplementary References

[S1] B. Zhao, Q. Liu, G. Wei, J. Wang, X.Y. Yu et al., Synthesis of $CoSe_2$ nanoparticles embedded in N-doped carbon with conformal TiO_2 shell for sodium-ion batteries. Chem. Eng. J. **378**(1), 122206 (2019). https://doi.org/10.1016/j.cej.2019.122206

[S2] G.D. Park, Y.C. Kang, One-pot synthesis of $CoSe_x$–rGO composite powders by spray pyrolysis and their application as anode material for sodium-ion batteries. Chem. Eur. J. **22**(12), 4140-4146 (2016). https://doi.org/10.1002/chem.201504398

[S3] S.K. Park, J.K. Kim, Y.C. Kang, Excellent sodium-ion storage performances of $CoSe_2$ nanoparticles embedded within N-doped porous graphitic carbon nanocube/carbon nanotube composite. Chem. Eng. J. **328**(1), 546-555 (2017). https://doi.org/10.1016/j.cej.2017.07.079

[S4] J. Yang, H. Gao, S. Men, Z. Shi, Z. Lin et al., $CoSe_2$ nanoparticles encapsulated by N-doped carbon framework intertwined with carbon nanotubes: high-performance dual-role anode materials for both Li- and Na-ion batteries. Adv. Sci. **5**(12), 1800763 (2018). https://doi.org/10.1002/advs.201800763

[S5] S.K. Park, Y.C. Kang, MOF-templated N-doped carbon-coated $CoSe_2$ nanorods supported on porous CNT microspheres with excellent sodium-ion storage and electrocatalytic properties. ACS Appl. Mater. Interfaces **10**(20), 17203-17213 (2018). https://doi.org/10.1021/acsami.8b03607

[S6] H. Guo, G. Liu, M. Wang, Y. Zhang, W. Li et al., In-situ fabrication of bone-like $CoSe_2$ nano-thorn loaded on porous carbon cloth as a flexible electrode for Na-ion storage. Chem. Asian J. **15**(9), 1493-1499 (2020). https://doi.org/10.1002/asia.202000189

[S7] Y. Fang, X.Y. Yu, X.W. Lou, Formation of hierarchical Cu-doped $CoSe_2$ microboxes via sequential ion exchange for high-performance sodium-ion batteries. Adv. Mater. **30**(21), 1706668 (2018). https://doi.org/10.1002/adma.201706668

[S8] S.H. Yang, S.K. Park, Y.C. Kang, Mesoporous $CoSe_2$ nanoclusters threaded with nitrogen-doped carbon nanotubes for high-performance sodium-ion battery anodes. Chem. Eng. J. **370**(1), 1008-1018 (2019). https://doi.org/10.1016/j.cej.2019.03.263

[S9] F. Kong, J. Wang, J. Chen, S. Tao, B. Qian et al., MOF-derived ultrasmall $CoSe_2$ nanoparticles encapsulated by an N-doped carbon matrix and their superior lithium/sodium storage properties. Chem. Commun. **56**(64), 9218-9221 (2020). https://doi.org/10.1039/d0cc03113e

[S10] M.H. Lee, S.J. Kim, D. Chang, J. Kim, S. Moon et al., Toward a low-cost high-voltage sodium aqueous rechargeable battery. Mater. Today **29**, 26-36 (2019). https://doi.org/10.1016/j.mattod.2019.02.004

[S11] D.A. Cristaldi, C.G. Fortuna, A. Gulino, A photoelectron spectroscopy study of lava stones. Anal. Methods **5**, 3458-3462 (2013). https://doi.org/10.1039/C3AY40136G

[S12] J. Fondard, E. Irisarri, C. Courrèges, M.R. Palacin, A. Ponrouch et al., SEI composition on hard carbon in Na-ion batteries after long cycling: influence of salts ($NaPF_6$, NaTFSI) and additives (FEC, DMCF). J. Electrochem. Soc. **167**, 070526 (2020). https://doi.org/10.1149/1945-7111/ab75fd

[S13] A. Ponrouch, D. Monti, A. Boschin, B. Steen, P. Johansson et al., Non-aqueous electrolytes for sodium-ion batteries. J. Mater. Chem. A **3**, 22-42 (2015). https://doi.org/10.1039/C4TA04428B

[S14] A. Darwiche, L. Bodenes, L. Madec, L. Monconduit, H. Martinez, Impact of the salts and solvents on the SEI formation in Sb/Na batteries: an XPS analysis. Electrochim. Acta **207**, 284-292 (2016). https://doi.org/10.1016/j.electacta.2016.03.089
